# Immunological and senescence biomarker profiles in patients after spontaneous clearance of hepatitis C virus: gender implications for long-term health risk

**DOI:** 10.1186/s12979-023-00387-z

**Published:** 2023-11-17

**Authors:** Rubén Martín-Escolano, Erick Joan Vidal-Alcántara, Javier Crespo, Pablo Ryan, Luis Miguel Real, Juan Ignacio Lazo-Álvarez, Joaquín Cabezas-González, Juan Macías, María Teresa Arias-Loste, Guillermo Cuevas, Ana Virseda-Berdices, Veronica Briz, Salvador Resino, María Ángeles Jiménez-Sousa, Amanda Fernández-Rodríguez

**Affiliations:** 1 Unidad de Infección Viral e Inmunidad, Centro Nacional de Microbiología, Instituto de Salud Carlos III (Campus Majadahonda), Carretera Majadahonda- Pozuelo, Km 2.2, Madrid, Majadahonda 28220 Spain; 2 Gastroenterology and Hepatology Department, Clinical and Traslational Research in Digestive Diseases, Valdecilla Research Institute (IDIVAL), Marqués de Valdecilla University Hospital, Santander, Spain; 3 Centro de Investigación Biomédica en Red en Enfermedades Hepáticas y Digestivas, Instituto de Salud Carlos III (ISCIII), Madrid, Spain; 4 Centro de Investigación Biomédica en Red en Enfermedades Infecciosas (CIBERINFEC), Instituto de Salud Carlos III (ISCIII), Madrid, Spain; 5 Internal Medicine Service, Hospital Universitario Infanta Leonor, Facultad de Medicina, Universidad Complutense de Madrid, Gregorio Marañón Health Research Institute, Madrid, Spain; 6 Instituto de Biomedicina de Sevilla (IBiS), Hospital Universitario Virgen de Valme Facultad de Medicina, Universidad de Sevilla, Seville, Spain; 7 Laboratory of Reference and Research On Viral Hepatitis, National Center for Microbiology, Institute of Health Carlos III, Madrid, Majadahonda Spain

**Keywords:** Biomarkers, HCV, Immune checkpoint proteins, SASP proteins, Spontaneous clearance

## Abstract

**Background:**

About 25% of patients with acute hepatitis C virus (HCV) infection show spontaneous clearance within the first six months of infection but may remain at risk of inflammaging, aging, and liver and non-liver disease complications. This study evaluated the differences in the plasma levels of immune checkpoints (ICs) and senescence-associated secretory phenotype (SASP) biomarkers between patients who had spontaneously eliminated HCV infection (SC group) and individuals without evidence of HCV infection (C group).

**Methods:**

We performed a multicenter retrospective study of 56 individuals: 32 in the SC and 24 in the C groups. ICs and SASP proteins were analyzed using a Luminex 200TM analyzer. The statistical analysis used Generalized Linear Models with gamma distribution (log-link) adjusted by significant variables and sex.

**Results:**

13 ICs (BTLA, CD137(4-1BB), CD27, CD28, CD80, GITR, HVEM, IDO, LAG-3, PD-1, PD-L1, PD-L2, and TIM-3) and 13 SASP proteins (EGF, Eotaxin, IL-1alpha, IL-1RA, IL-8, IL-13, IL-18, IP-10, SDF-1alpha, HGF, beta-NGF, PLGF-1, and SCF) were significantly higher in SC group after approximately more than two years of HCV clearance. After stratifying by sex, differences remained significant for males, which showed higher levels for 13 ICs and 4 SASP proteins in SC. While only PD-L2 was significantly higher in SC women, and no differences in SASP were found.

**Conclusions:**

Higher plasma levels of different IC and SASP proteins were found in individuals after more than two years of HCV clearance, mainly in men. Alterations in these molecules might be associated with an increased risk of developing liver and non-hepatic diseases.

**Supplementary Information:**

The online version contains supplementary material available at 10.1186/s12979-023-00387-z.

## Background

Hepatitis C virus (HCV) infection is a major global health issue, being a risk factor for liver fibrosis, cirrhosis, hepatic decompensation, hepatocellular carcinoma (HCC), and death [1]. About 25% of individuals show spontaneous clearance (SC) during acute HCV infection within the first six months [2]. Factors associated with higher HCV spontaneous resolution rates are known, such as some host and viral factors, including *interleukin (IL) 28* CC genotype [2], young age [3], female sex [4], and HCV genotype 1 [2], among others. However, the risk of inflammation-induced aging (inflammaging), aging, and disease complications related to HCV infection after SC has not been thoroughly investigated. Knowledge of HCV clearance is limited due to the typically asymptomatic nature of the initial infection and the highly marginalized nature of specific at-risk populations, such as intravenous drug users. This circumstance results in a limited number of studies in people who have experienced SC, due to the small number of cases identified. Studying the HCV footprint in patients after SC will improve knowledge of the sequelae of an acute HCV infection in hosts and determine if these individuals require closer monitoring after HCV clearance.

Both acute and chronic HCV infections induce ROS generation, DNA damage, telomere shortening, epigenetic modifications of DNA and histones, upregulation of several immune checkpoint (IC) proteins, and induction of senescence-associated secretory phenotype (SASP) [5, 6]. The upregulation of several IC proteins can drive effector immune T cells into a state known as “exhaustion” [6, 7]. This state is characterized by sustained expression of ICs, reduced T cell effector function, and poor recall responses, leading to limited HCV clearance, the persistence of HCV, and disease progression related to hepatitis C. In addition, this “exhaustion” can itself drive immune-related adverse events (irAEs), which have proven to be difficult to predict, both in severity and timing [8, 9]. In regards to SASP, it is characterized by sustained expression of a combination of inflammatory factors such as several ILs, proteases, and growth factors, among others, [10] leading to the development of long-term sequelae such as chronic autoimmune disorders and cancers [5, 11]. However, there are no previous studies describing the long-term impact on the IC and SASP proteins in patients who spontaneously cleared HCV infection in the past.

This study evaluated the differences in IC and SASP biomarkers plasma levels between patients who had spontaneously eliminated HCV infection more than two years previously and a control group with no evidence of prior HCV infection.

## Results

### Patient characteristics

Characteristics of the 56 individuals stratified by previous HCV infection status (SC and C groups) are presented in Table 1. The median age was 53, 48.2% were males (biological sex and gender were coincident in all individuals), and BMI was 25.3 kg/m^2^, with no significant differences between the groups, although we lack complete clinical information for all individuals. Regarding the *IFN-λ*_*4*_ genotype (rs12979860), 58.2% of individuals showed a favorable CC genotype. The SC group showed significantly higher values of AST (*p*-value = 0.004), ALT (*p*-value = 0.011), FIB4 (*p*-value = 0.001), and APRI (*p*-value = 0.001) indexes (Table 1). The median time since HCV clearance was three years. The characteristics of men (*n* = 27) and women (*n* = 29) are displayed in Table 2. No statistically significant differences were found for age, BMI, and *IFN*-*λ*_*4*_ genotype (rs12979860) for both male and female individuals. Regarding liver and metabolic markers, the SC group showed significantly higher FIB4 and APRI indices values for both males and females. AST was also significantly higher in the SC male group.

**Table 1:** Clinical and epidemiological characteristics of 56 individuals stratified by HCV infection status

	**All**	**SC**	**C**	***p***
**No**	56	32 (57.1%)	24 (42.9%)	
Age (years)	53 (47–59)	55 (47–60)	51 (47–58)	0.289
Sex (male)	27 (48.2%)	16 (50.0%)	11 (45.8%)	0.969
BMI (kg/m^2^) (*n* = 34)	25.3 (22.8–28.0)	25.0 (23.2–27.5)	25.3 (22.5–28.4)	0.985
Smoker (*n* = 22)				–
Never	–	–	11 (50.0%)	
Previous (> 6 months)	–	–	7 (31.8%)	
Current	–	–	4 (18.2%)	
Route of transmission (*n* = 9)				–
Intravenous drug user	–	6 (66.7%)	–	
Medical and Aesthetic procedures	–	2 (22.2%)	–	
Work accident	–	1 (11.1%)	–	
Time from clearance (years) (*n* = 23)	–	3.0 (2.0–11.5)	–	–
*IFN-λ*_*4*_ genotype (rs12979860) (*n* = 55)				0.802
CC	32 (58.2%)	19 (61.3%)	13 (54.2%)	
CT	18 (32.7%)	9 (29.0%)	9 (37.5%)	
TT	5 (9.1%)	3 (9.7%)	2 (8.3%)	
Stage of liver fibrosis (*n* = 9)				–
F0	–	1 (11.1%)	–	
F1	–	7 (77.8%)	–	
F2	–	1 (11.1%)	–	
F3	–	0 (0.0%)	–	
F4	–	0 (0.0%)	–	
**Liver markers**				
FIB4 (*n* = 46)	1.2 (1.0–1.5)	1.4 (1.2–2.0)	1.0 (0.8–1.2)	**0.001**
APRI (*n* = 48)	0.3 (0.2–0.4)	0.3 (0.3–0.4)	0.2 (0.2–0.3)	**0.001**
AST (mg/dL) (*n* = 54)	21.0 (16.3–29.5)	24.0 (18.5–36.5)	19.0 (15.0–21.0)	**0.004**
ALT (mg/dL) (*n* = 54)	19.5 (15.0–31.8)	25.5 (17.8–34.5)	15.0 (13.0–21.8)	**0.011**
GGT (mg/dL) (*n* = 53)	22.0 (14.0–39.0)	24.0 (16.0–56.0)	21.0 (13.3–30.3)	0.138
**Metabolic syndrome markers**				
TyG (*n* = 46)	8.3 (8.1–8.7)	8.4 (8.2–8.7)	8.3 (8.0–8.7)	0.542
METS IR (*n* = 32)	33.1 (27.9–41.2)	32.8 (29.2–36.1)	33.4 (27.8–42.2)	0.742

Statistics: The values are expressed as the absolute number (percentage) and median (interquartile range). *p*-values were calculated by the Chi-square test and the Mann–Whitney U test

*Abbreviations*: *ALT* alanine transaminase, *APRI* AST to platelet ratio index, *AST* aspartate transaminase, *BMI* body mass index, *C* control, *FIB4* fibrosis-4 index, *GGT* gamma-glutamyl transferase, *HOMA IR* homeostatic model assessment for insulin resistance, *METS IR* metabolic score for insulin resistance, *SC* spontaneous clearance, *TyG* triglyceride glucose index

**Table 2:** Clinical and epidemiological characteristics of 27 male and 29 female individuals stratified by HCV infection status

	**Male**				**Female**			
	**All**	**SC**	**HC**	***p***	**All**	**SC**	**HC**	***p***
**No**	27	16 (59.3%)	11 (40.7%)		29	16 (37.2%)	13 (30.2%)	
Age (years)	53 (49–58)	55 (51–59)	51 (48–54)	0.373	52 (46–60)	54 (47–62)	49 (44–59)	0.496
BMI (kg/m^2^) (*n* = 16)	26.4 (25.4–30.6)	25.6 (24.2–26.9)	26.4 (25.8–30.8)	0.441	22.9 (21.1–25.7)	23.0 (20.2–27.8)	22.8 (21.3–24.4)	0.999
Smoker (*n* = 9)				–				–
Never	–	–	3 (33.3%)		–	–	8 (61.5%)	
Previous (> 6 months)	–	–	5 (55.6%)		–	–	2 (15.4%)	
Current	–	–	1 (11.1%)		–	–	3 (23.1%)	
Route of transmission (*n* = 3)				–				–
Intravenous drug user	–	3 (100.0%)	–		–	3 (50.0%)	–	
Medical and Aesthetic procedures	–	0 (0.0%)	–		–	2 (33.3%)	–	
Work accident	–	0 (0.0%)	–		–	1 (16.7%)	–	
Time from clearance (years) (*n* = 14)	–	2.3 (1.3–3.8)	–	–	–	9.0 (4.0–28.0)	–	–
*IFN-λ*_*4*_ genotype (rs12979860)				0.589				0.363
CC	17 (63.0%)	11(68.8%)	6 (54.5%)		15 (53.6%)	8 (53.3%)	7 (53.8%)	
CT	7 (25.9%)	4 (25.0%)	3 (27.3%)		11 (39.3%)	5 (33.3%)	6 (46.2%)	
TT	3 (11.1%)	1 (6.2%)	2 (18.2%)		2 (7.1%)	2 (13.3%)	0 (0.0%)	
Stage of liver fibrosis (*n* = 3)				–				–
F0	–	1 (33.3%)	–		–	0 (0.0%)	–	
F1	–	2 (66.7%)	–		–	5 (83.3%)	–	
F2	–	0 (0.0%)	–		–	1 (16.7%)	–	
F3	–	0 (0.0%)	–		–	0 (0.0%)	–	
F4	–	0 (0.0%)	–		–	0 (0.0%)	–	
**Liver markers**								
FIB4 (*n* = 21)	1.2 (1.0–1.4)	1.3 (1.2–2.0)	1.0 (0.9–1.1)	**0.006**	1.2 (0.9–1.5)	1.5 (1.0–1.6)	1.0 (0.8–1.2)	**0.038**
APRI (*n* = 21)	0.3 (0.2–0.4)	0.4 (0.3–0.5)	0.2 (0.2–0.3)	**0.020**	0.3 (0.2–0.3)	0.3 (0.2–0.4)	0.2 (0.2–0.3)	**0.023**
AST (mg/dL) (*n* = 25)	24.0 (19.0–33.0)	28.0 (24.0–38.5)	19.0 (15.5–22.3)	**0.008**	19.0 (16.0–24.0)	21.5 (16.0–27.8)	19.0 (15.0–20.0)	0.187
ALT (mg/dL) (*n* = 26)	27.0 (17.3–37.5)	28.5 (22.5–40.8)	19.0 (14.0–25.5)	0.065	17.5 (13.8–25.0)	19.0 (17.0–28.8)	15.0 (11.8–17.5)	0.053
GGT (mg/dL) (*n* = 25)	28.0 (22.0–39.0)	30.0 (22.0–56.0)	28.0 (26.0–33.3)	0.803	15.5 (11.0–34.0)	19.5 (13.3–54.8)	13.5 (10.8–17.8)	0.148
**Metabolic syndrome markers**								
TyG (*n* = 21)	8.5 (8.2–8.7)	8.5 (8.1–8.6)	8.7 (8.4–8.9)	0.173	8.2 (7.9–8.5)	8.4 (8.3–8.7)	8.1 (7.9–8.2)	0.060
METS IR (*n* = 14)	37.1 (33.7–46.0)	33.7 (32.6–39.7)	40.3 (36.8–46.0)	0.454	28.1 (25.9–34.6)	29.2 (24.4–36.1)	28.0 (26.0–30.1)	0.849

Statistics: The values are expressed as the absolute number (percentage) and median (interquartile range). *p*-values were calculated by the Chi-square test and the Mann–Whitney U test

*Abbreviations*: *ALT* alanine transaminase, *APRI* AST to platelet ratio index, *AST* aspartate transaminase, *BMI* body mass index, *C* control, *FIB4* fibrosis-4 index, *GGT* gamma-glutamyl transferase, *HCV* hepatitis C virus, *HCV-RNA* viral load of hepatitis C, *HOMA IR* homeostatic model assessment for insulin resistance, *METS IR* metabolic score for insulin resistance, *SC* spontaneous clearance, *TyG* triglyceride glucose index

The biochemical characteristics of the SC and C groups are shown in Additional file 1. Biochemical data was also stratified by sex.

### ICs biomarkers comparison between SC and C groups

**Fig. 1:**
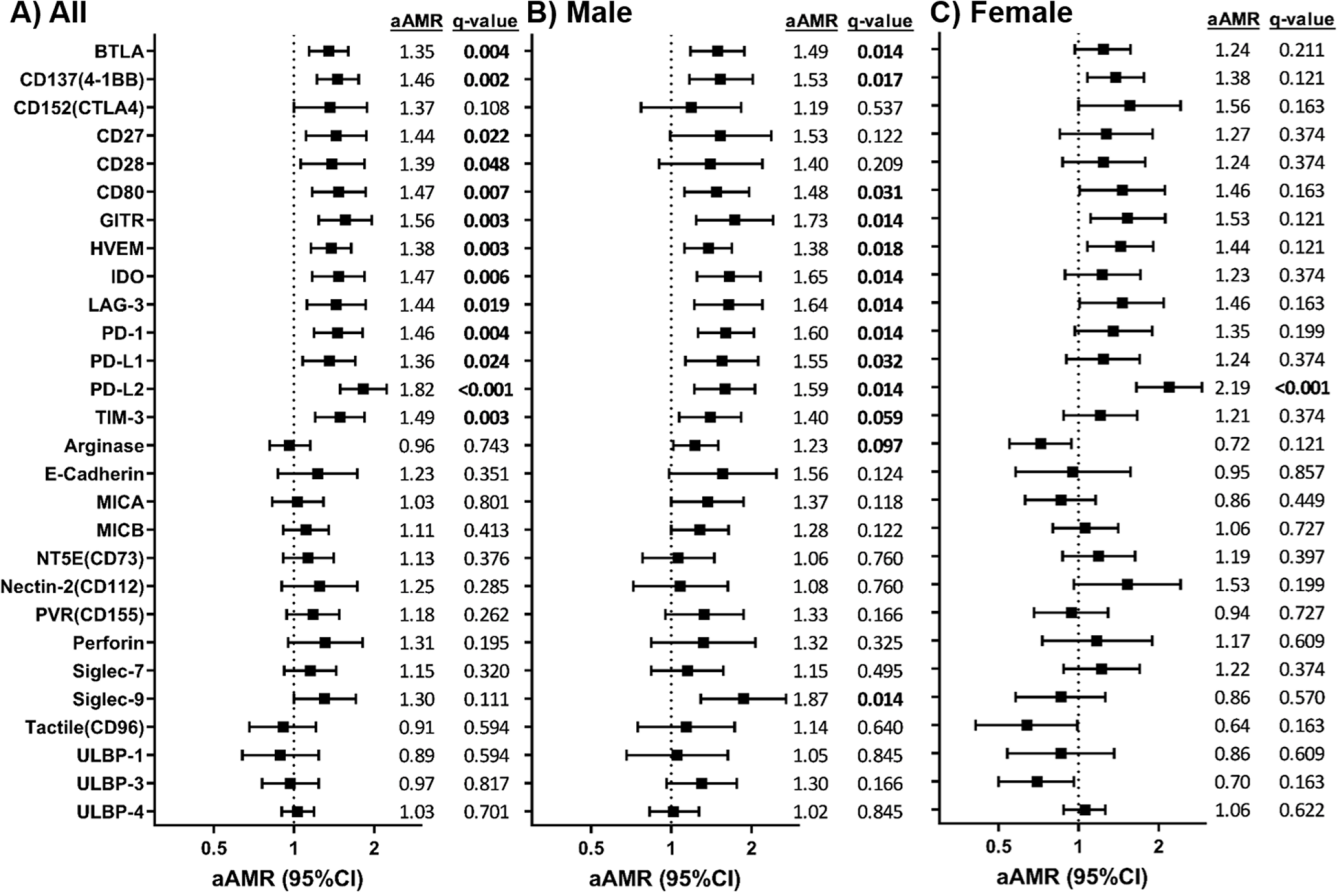
Comparison of plasma immune checkpoints between subjects who spontaneously cleared HCV versus the control group: **A** All individuals, **B** Male individuals, **C** Female individuals. Statistics: Data were calculated by Generalized Linear Models (GLM) with a gamma distribution (log-link) adjusted by those clinical variables selected following a stepwise method from age, sex, *IFN-λ*_*4*_ genotype (rs12979860), and aspartate transaminase (AST) for **A**), and by sex, *IFN-λ*_*4*_ genotype (rs12979860), and aspartate transaminase (AST) for **B**) and **C**), (see Results Section). The q-values represent *p*-values corrected for multiple testing using the False Discovery Rate (FDR). Significant differences are shown in bold. Abbreviations: AMR, arithmetic mean ratio; aAMR, adjusted AMR; 95%CI, 95% of confidence interval; q, corrected level of significance; BTLA, B and T lymphocyte attenuator; CD, cluster of differentiation; GITR, glucocorticoid-induced TNFR-related; HVEM, herpesvirus entry mediator; IDO, indoleamine 2,3-dioxygenase; LAG-3, lymphocyte activation gene-3; PD-1, programmed cell death protein 1; PD-L1, programmed death-ligand 1; PD-L2, programmed death-ligand 2; TIM-3, T-cell immunoglobulin and mucin-domain containing-3; MICA, MHC class I chain-related gene A; MICB, MHC class I chain-related gene B; NT5E, ecto-5′-nucleotidase; PVR, poliovirus receptor; Siglec, sialic acid-binding immunoglobulin-type lectin; ULBP, human ligand for binding protein

Adjusted GLM models showed significantly higher levels of 13 ICs proteins in the SC group compared to C group (Fig. 1A; full description in Additional file 2): BTLA (aAMR = 1.35; q = 0.004), CD137(4-1BB) (aAMR = 1.46; q = 0.002), CD27 (aAMR = 1.44; q = 0.022), CD28 (aAMR = 1.39; q = 0.048), CD80 (aAMR = 1.47; q = 0.007), GITR (aAMR = 1.55; q = 0.003), HVEM (aAMR = 1.38; q = 0.003) IDO (aAMR = 1.47; q = 0.006), LAG-3 (aAMR = 1.44; q = 0.019), PD-1 (aAMR = 1.46; q = 0.004), PD-L1 (aAMR = 1.36; q = 0.024), PD-L2 (aAMR = 1.82; q < 0.001), and TIM-3 (aAMR = 1.49; q = 0.003).

After stratifying by sex, adjusted GLM models for males showed significantly higher levels of 13 IC proteins in the SC group compared to the C group (Fig. 1B; full description in Additional file 3), 11 of which were common with the comparison of all groups: BTLA (q = 0.014), CD137(4-1BB) (q = 0.017), CD80 (q = 0.031), GITR (q = 0.014), HVEM (q = 0.018) IDO (q = 0.014), LAG-3 (q = 0.014), PD-1 (q = 0.014), PD-L1 (q = 0.032), PD-L2 (q = 0.014), TIM-3 (q = 0.059); and two of them, were only significant for the male comparison: arginase (q = 0.097), and Siglec-9 (q = 0.014). For females, only PD-L2 (q < 0.001) showed significantly higher levels in the SC group compared to the C group (Fig. 1C; full description in Additional file 4).

### SASP biomarkers comparison between SC and C groups

**Fig. 2:**
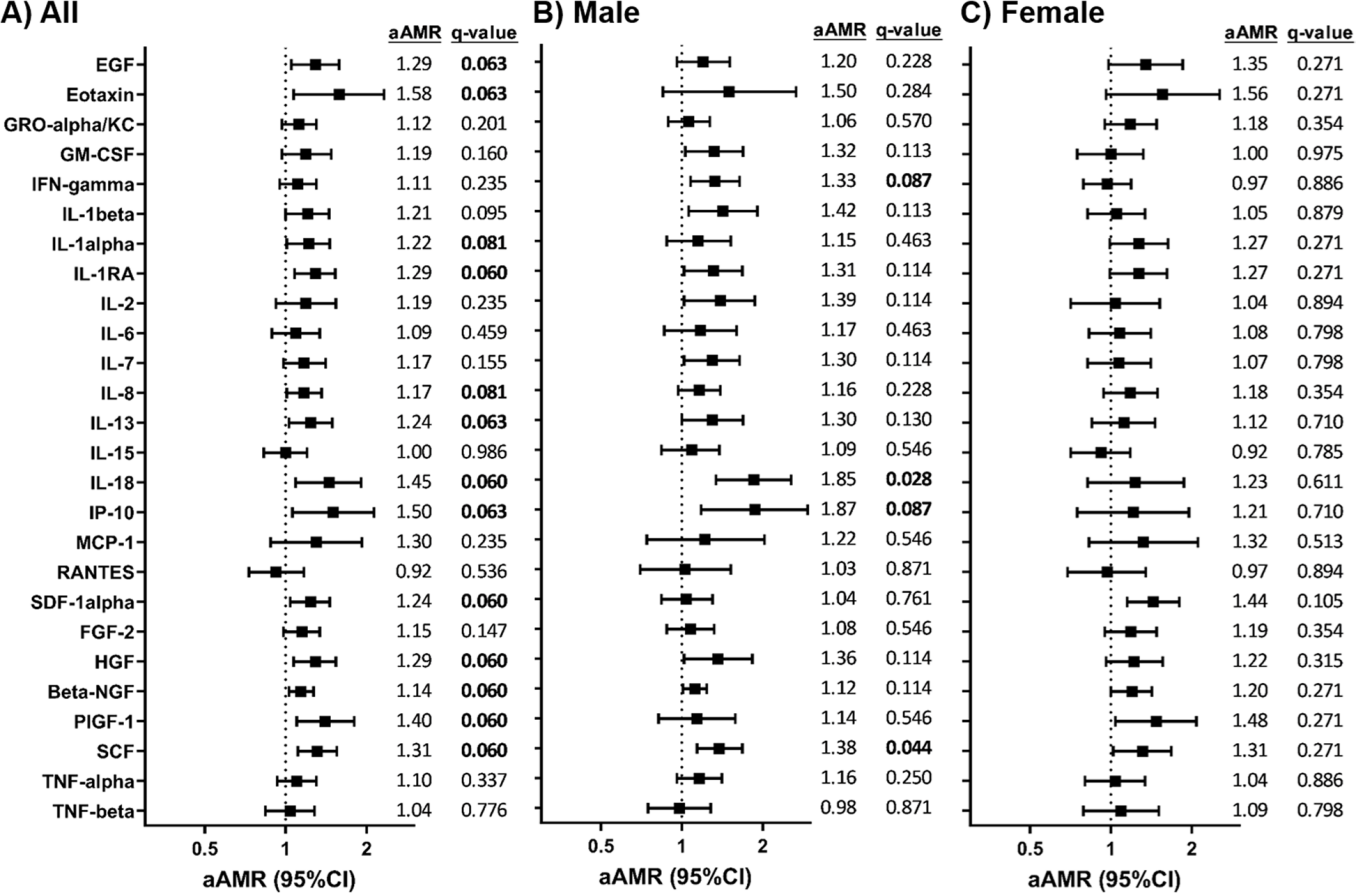
Comparison of senescence-associated secretory phenotype (SASP) proteins between subjects who spontaneously cleared HCV versus the control group: **A** All individuals, **B** Male individuals, **C** Female individuals. Statistics: Data were calculated by Generalized Linear Models (GLM) with a gamma distribution (log-link) adjusted by those clinical variables selected following a stepwise method from age, sex, *IFN-λ*_*4*_ genotype (rs12979860), and aspartate transaminase (AST) for A), and by sex, *IFN-λ*_*4*_ genotype (rs12979860), and aspartate transaminase (AST) for B) and C)(see Results Section). The q-values represent *p*-values corrected for multiple testing using the False Discovery Rate (FDR). Significant differences are shown in bold. Abbreviations: AMR, arithmetic mean ratio; aAMR, adjusted AMR; 95%CI, 95% of confidence interval; q, corrected level of significance; EGF, epidermal growth factor; GRO-alpha/KC, chemokine growth-regulated protein alpha; GM-CSF, granulocyte–macrophage colony-stimulating factor; IFN, interferon; IL, interleukin; MCP-1, C–C motif chemokine ligand 2; RANTES, C–C motif chemokine ligand 5; SDF-1alpha, stromal cell-derived factor 1alpha; FGF-2, fibroblast growth factor 2; HGF, hepatocyte growth factor; Beta-NGF, nerve growth factor β; PLGF-1, placental growth factor; SCF, skp, cullin, F-box containing complex; TNF, tumoral necrosis factor

Adjusted GLM models showed significantly higher levels of 13 SASP proteins in the SC group compared to the C group (Fig. 2A; full description in Additional file 5): EGF (aAMR = 1.29; q = 0.063), eotaxin (aAMR = 1.58; q = 0.063), IL-1alpha (aAMR = 1.22; q = 0.081), IL-1RA (aAMR = 1.29; q = 0.060), IL-8 (aAMR = 1.17; q = 0.081), IL-13 (aAMR = 1.24; q = 0.063), IL-18 (aAMR = 1.45; q = 0.060) IP-10 (aAMR = 1.50; q = 0.063), SDF-1alpha (aAMR = 1.24; q = 0.060), HGF (aAMR = 1.29; q = 0.060), beta-NGF (aAMR = 1.14; q = 0.060), PLGF-1 (aAMR = 1.40; q = 0.060), and SCF (aAMR = 1.31; q = 0.060).

After stratifying by sex, adjusted GLM models for males showed significantly higher levels of four SASP proteins in the SC group compared to the C group (Fig. 2B; full description in Additional file 6): IFN-gamma (q = 0.087), IL-18 (q = 0.028), IP-10 (q = 0.087), and SCF (q = 0.044). Except for IFN-gamma, the rest of the SASP proteins were also significant in all individuals. For females, no significant differences in SASP levels were found between the SC and C groups (Fig. 2C; full description in Additional file 7).

**Fig. 3:**
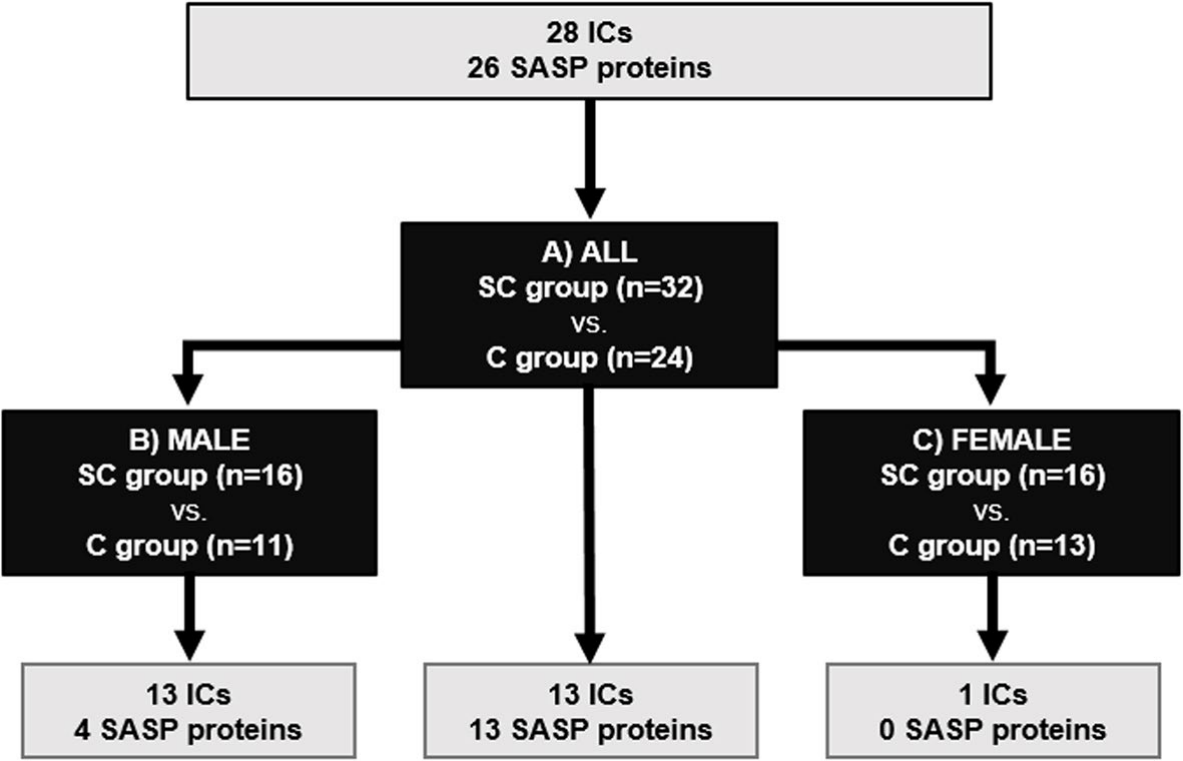
Study design flowchart. Significant immune checkpoints (ICs) and senescence-associated secretory phenotype (SASP) proteins between subjects who spontaneously cleared HCV versus the control group are shown: A) All individuals, B) Male individuals, C) Female individuals. Statistics: Data were calculated by Generalized Linear Models (GLM) with a gamma distribution (log-link) adjusted by those clinical variables selected following a stepwise method from age, sex, *IFN-λ*_*4*_ genotype (rs12979860), and aspartate transaminase (AST) for A), and by sex, *IFN-λ*_*4*_genotype (rs12979860), and aspartate transaminase (AST) for B) and C), (see Results Section). Abbreviations: ICs, immune checkpoints; SASP, senescence-associated secretory phenotype; SC, spontaneous clearance; C, control

Figure 3 summarizes the comparison groups and the significant results obtained for the GLM statistical analysis of IC and SASP proteins.

### ICs and SASP biomarkers comparison between males and females within SC and C groups

For both SC and C groups, adjusted GLM models showed a trend to lower ICs levels in males compared to females, but no significant differences were found for the SC group; E-Cadherin (aAMR = 0.44; q-value = 0.005) and Siglec-9 (aAMR = 0.48; q = 0.005) were significant for C group (Additional file 8). For SASP proteins, GLM models showed no significant differences between males vs. females within SC and C groups (Additional file 9).

### Correlation analysis between IC and SASP proteins

Since most of the statistically significant differences for IC/SASP proteins between SC and C groups were found in men, we studied the correlation between IC and SASP proteins in this subgroup (Additional file 10). Significant positive correlations were found for most IC with SASP proteins (Additional file 10A). When stratified by HCV infection status, males who spontaneously cleared HCV infection also showed many significant positive IC-SASP correlations (Additional file 10B). In contrast, fewer significant positive correlations were found for men in the C group (Additional file 10C).

## Discussion

This study describes higher plasma levels of 13 IC and 13 SASP biomarkers in individuals who spontaneously cleared HCV infection after a long period since HCV infection (more than two years) compared to a control group without evidence of previous HCV infection. Additionally, we observed a clear sex bias as all differences were mainly found in men, either between or within study groups. This immune dysregulation might be associated with an increased risk of developing liver and non-hepatic diseases in the future, especially in men.

HCV infection is a potent inducer of ICs and SASP proteins, which increase during acute and chronic HCV infection [5, 6]. The sustained elevation of ICs and SASP proteins induces a state of inflammaging and immunosenescence, respectively [12–15], which may persist as residual effects after HCV clearance [16, 17]. In addition, it is reasonable to assume that inflammaging and immunosenescence also occur in extrahepatic locations. Thus HCV patients might be at risk of developing diseases such as kidney disease, cardiovascular disease (CVD), cancer, and irAEs, among others [5, 8, 9, 11].

Following chronic HCV infection, many studies have shown that only partial restoration of many immune functions is achieved after HCV clearance with DAAs [17–20], underscoring the importance of monitoring the status of inflammaging and immunosenescence in patients during and after chronic HCV infection. However, there is limited information on the long-term impact of acute HCV infection. Our results showed that individuals who experienced spontaneous HCV clearance showed higher plasma levels of ICs than those without evidence of previous HCV infection, and these levels remained elevated for more than two years following HCV clearance. The increase of IC levels is a mechanism to negatively regulate the magnitude and duration of effector T cells following an acute viral infection. IC levels are downregulated following viral clearance [21, 22]; however, there is little information on whether the levels normalize over time after SC. In this context, our data suggest that they remain elevated long after HCV clearance, which could be associated with T-cell exhaustion.

These results align with previous studies in chimpanzee models, where PD-1 on HCV-specific CD8 + T cells showed sustained expression as late as seven years after spontaneous viral clearance [23]. In addition, PD-1 expression in HCV-specific CD4 + T cells showed increased levels in either HCV acute, chronic or spontaneous clearers individuals compared to individuals who have never been infected, being specially higher during acute and chronic infection [24]. Other ex-vivo studies have also observed increased PD-1 levels in HCV-specific CD4 + T cells after the elimination of chronic HCV infection [25]. Alternatively, it has been described that BTLA exhibits high expression in patients irrespective of their HCV infection status (acute, chronic, or spontaneously resolved), without significant differences among them [24]. Ex-vivo studies have observed that HCV-specific CD4 + T cells increased BTLA expression during the chronic phase of the HCV infection, maintaining high levels during the course of therapy with DAAs [26]. These ex-vivo studies have been focused on the expression levels of surface HCV-specific CD4 + T cells, lacking a global view of the whole organism. However, our study analysed the protein levels in plasma, reflecting the expression levels of different cell populations as a whole. Even so, our results are in line with previous studies in HCV-specific CD4 + T cells.

Our results indicate that BTLA remains elevated for more than two years following HCV clearance.

On the other hand, viral-induced senescence has been previously described for various viruses as a mechanism to limit viral replication [27]. However, little is known about its behavior after HCV resolution. Senescent cells secrete SASP proteins, producing a pro-inflammatory and pro-proliferative microenvironment that might contribute to liver disease progression and associated complications [28]. We observed that SASP proteins remained elevated after a long-time following resolution of HCV. The increase of these senescence markers has been mainly studied in chronic hepatitis C patients, where they increase with fibrosis progression [28]. But to our knowledge, there was no previous knowledge of plasma SASP levels in SC individuals. Our results showed 13 SASP proteins elevated in the SC group, such as eotaxin, a pro-inflammatory cytokine associated with liver fibrosis progression [29]. IP-10 (also known as CXCL10) – which stimulates monocytes, natural killer, and causes T-cell migration – is increased in patients with tissue damage and extrahepatic manifestations [29–31]. Thus, the senescence profile seems to be only partially restored two years after spontaneous viral clearance, similar to the observed after HCV treatment [32]. Therefore, the increased levels of ICs and SASP proteins observed in individuals who spontaneously cleared HCV infection after a long period could be potential biomarkers for future comorbidities or HCV-related diseases in these individuals.

Interestingly, our data revealed a substantial sex bias as all differences were predominantly observed in men. Therefore, male individuals who spontaneously cleared HCV maintained higher levels of both ICs and SASP proteins after a long period since HCV clearance, suggesting a higher risk of future liver and non-liver disease complications. Males usually show higher viral load, reduced viral clearance, and increased liver fibrosis progression [33]. In our study, we observed a higher prevalence of drug users in males, but the route of transmission was documented only in a small proportion of patients (less than 30% of the individuals), therefore this variable could not be further explored. Sex-based differences in immune responses result from a complex interaction between sex-specific genes, environment, and hormonal milieu. However, the effect of sex on ICs expression and function is still unknown. We found higher plasma levels of arginase, siglec-9, and IFN-gamma that were significant exclusively in males. Arginase, a driver for immune suppression since it catabolizes arginine, an amino acid required for T cell activation and proliferation [34], is increased in chronic hepatitis C patients with cirrhosis, chronic liver disease, and HCC [35]. IFN-gamma, an inflammatory cytokine that plays an essential role in modulating the immune response, has also been increased in chronic hepatitis C patients, which might lead to inflammaging, fibrosis, and autoimmune diseases [36].

Limited data is available after acute HCV infection, but HCV-induced changes may persist after viral eradication, and some senescence changes in SC individuals may persist a substantial duration after HCV resolution, similar to chronic hepatitis C patients, where some changes seem irreversible [37].

On the other hand, we did not observe differences between female SC and control individuals for SASP markers and only one for ICs, suggesting a reduced risk of developing long-term sequelae due to an acute HCV infection. The sex bias may be attributed to sex-based differences in immunity [2], which result in women experiencing a lower burden of infections [38], as well as a higher prevalence of several autoimmune diseases [38]. Most of these differences have a genetic origin as the X-chromosome encodes essential genes in modulating innate and adaptive immunity. Additionally, sex hormones such as estrogen are protective against genotoxic stress and SASP-related inflammation [39]. In this regard, PD-L2 was the only IC significantly different between SC and C females, whose plasma levels were even higher than SC males. PD-L2, the second ligand of PD-1, is involved in dampening the host immune response [40] plays essential role in autoimmune diseases, cancer, and chronic viral infections. A previous report has identified an elevated expression of PD-L2 in hepatocytes infected by HCV [41]. Thus, the increase of PD-L2 expression in women responding to HCV acute infection could be one key factor in HCV clearance and remain elevated over the long term. The implications of an elevated PD-L2 in plasma are unclear, but some reports point to a better prognostic value in different cancers.

Additionally, we explored the correlation between ICs and SASP markers profiles in HCV infection. Viral infections are one of the most important drivers of premature aging [5, 42], known as viral-induced senescence (VIS). Both inflammaging and immunosenescence processes are involved in a vicious cycle that impairs the functioning of the aging immune system. On the one hand, senescent cells are characterized by a pro-inflammatory profile, leading to inflammaging, which drives altered adaptive immune responses, contributing to immunosenescence [12–15]. Our results support this, as we observed significant positive correlations between most ICs and SASP proteins, such as IFN-gamma and IL-18, which showed a larger number of significant positive correlations with ICs. During viral infections, IL-18 [43] promotes T-cell activation, which is vital for HCV clearance. These cells secrete pro-inflammatory cytokines such as IFN-gamma [44] that can promote long-term stimulation leading to T cell exhaustion after HCV infection [45]. T-cell exhaustion can also be caused by long-term overexpression of ICs to modulate pro-inflammatory cytokine secretion and normalize immune function. Therefore, as we observed higher plasma levels of ICs and SASP in male SC individuals, these individuals might remain at risk of developing immune-metabolic events. Further studies should be developed in the future to associate these alterations with the possible development of metabolic associated fatty liver disease (MAFLD), especially in men.

We have an insufficient knowledge about the transmission pathways of most patients. Since the SC group was obtained from a population-based HCV screening in general population, clinical data on the acute phase or additional information such as the route of transmission were not available (unknown) for most of the individuals. An additional source of senescence is drug abuse [46], which can strongly contribute to a senescence accelerating factors. As we have an insufficient knowledge about the transmission pathways of most patients, drug abuse would be a source of heterogeneity not considered. Drug use also interferes with the immune response mediated by antibodies and decreases the production of ILs and IFN-γ, although different effects are observed depending of the type of substance [47, 48]. In the case of cocaine, multiple negative effect on immune system have been described, such as the decrease in cytokine formation, lymphocyte proliferation, natural killer cell activity and antibody formation, among others [49]. Other drugs, such as morphine [50], heroin [51] or methamphetamine [52], destabilize IFN-mediated innate immunity. In any case, drug use inhibits host immunity, which is related with an increased susceptibility to infections [47, 48]. To our knowledge, there are no long-term studies on the potential impact of previous drug abuse on the immune system and neither on an accelerated senescence, which remain uncertain but cannot be discarded. Thus, as we have no information about the transmission pathways of most patients due to they did not report a possible route of infection, our results should be interpreted with caution. More studies would be needed to corroborate our findings and evaluate their long-term impact on SC patients.

Our results could be valuable for the design of a controlled human infection model (CHIM) for HCV vaccine development, which requires careful consideration [53]. In the absence of an immune competent animal model for HCV, this concept proposes to deliberately infect healthy human volunteers with HCV for testing the efficacy of candidate vaccines [54, 55], to accelerate development, reduce costs, and allow the selection of more promising candidates, among others. However, scarce data are available about the impact of an acute HCV infection in general population, thus although mortality rate is comparable [56], extensive studies regarding additional complications such as cancer or senescence-related events have not been approached yet.

Therefore, our study could be borne in mind for the use of CHIM studies, as HCV-related clinical sequelae may not only occur years after HCV chronic infection. For that reason, before HCV CHIM become real, additional longitudinal studies should be performed in SC individuals to confirm the absence of long-term harm associated with HCV self-limited infection.

To correctly interpret our data, note that this is a preliminary study with a limited sample size, which might have limited the possibility of finding additional statistical significance differences of reduced estimates. This is a retrospective study that may have introduced biases. However, we controlled for the most relevant variables by including them as covariates in the GLM models. We have applied the FDR correction to limit false-positive results.

Additional putative confounders related to senescence such as diet or physical activity could not be recorded. Therefore, we could not control for these variables by including them as covariates in the GLM. In addition, the cohort is homogeneous, excluding individuals with advanced fibrosis, hepatic decompensation, alcohol-induced liver injury, active HBV or HIV coinfection, opportunistic infections, and other concomitant diseases such as diabetes, neoplasia, or autoimmune diseases.

## Conclusions

Higher plasma levels of different IC and SASP proteins were found in individuals after a long time from HCV clearance, compared to the control group. These differences were mainly found in men, whose alterations might be associated with an increased risk of developing liver and non-hepatic diseases.

## Methods

### Study subjects

We conducted a multicenter retrospective study across three Public Spanish Hospitals in Spain: Hospital Universitario Virgen de Valme (Sevilla), Hospital Universitario Marqués de Valdecilla (Santander), and Hospital Universitario Infanta Leonor (Madrid). Fifty-six individuals were included, of which: i) 32 individuals were acutely infected with HCV and experienced SC after HCV infection with at least six months of follow-up since diagnosis from 2014 to 2017 (SC group: undetectable HCV-RNA viral load and positive HCV antibodies); and ii) 24 individuals with no evidence of prior HCV infection voluntarily recruited from the Centro Nacional de Microbiología (CNM) (Madrid) (C group: negative HCV antibodies).

The SC individuals were recruited in 2016 for a population-based HCV screening in general population [57]. Subjects with positive anti-HCV and negative PCR were invited to self-report a brief questionnaire and to take a biological sample. Individuals who met inclusion criteria, provided written informed consent, and had an available plasma sample were included in the study.

Exclusion criteria were: i) individuals below 18 years old; ii) previous HCV treatment; iii) hepatitis B virus (HBV) or HIV active infection; iv) active drug or alcohol addiction, and v) clinical evidence of hepatic decompensation, alcohol-induced liver injury, opportunistic infections, and other concomitant diseases such as diabetes, neoplasia, or autoimmune disease, among others.

The study was approved by the Research Ethics Committee of the Institute of Health Carlos III (CEI PI 11_2015-V4) and was conducted following the Declaration of Helsinki. All participants signed a written consent to participate in the study.

### Clinical data and samples

Clinical and epidemiological characteristics were collected from medical records. The liver stiffness measurement (LSM) was assessed by transient elastometry (FibroScan®, Echosens, Paris, France) and expressed in kilopascals (kPa). Subjects were stratified according to cut-offs of LSM: < 7.1 kPa (F0–F1: absence or mild fibrosis) and 7.1–9.4 kPa (F2: significant fibrosis). The time between acute infection and clearance, was estimated from the first year they reported risk behaviours, such as sharing needles and other injection paraphernalia in those individuals with a history of intravenous drug use (IDU), sexual risk behaviour, blood transfusion or medical procedure, among others.

Peripheral blood samples were collected in ethylenediaminetetraacetic acid tubes and processed within 4 h of extraction. Plasma was clarified and stored at -80ºC until samples were transferred to the National Center for Microbiology for subsequent analysis.

### Outcome variable

The outcome variable was the HCV infection status (SC group versus C group) and sex. Additionally, sex were confirmed by PCR as previously described by [58]. Primer sequences and PCR conditions were included in Additional file 11.

### Multiplex immunoassays

Immuno-Oncology Checkpoint Human ProcartaPlex™ Panel 1 and Panel 2 (Invitrogen™) were used to measure 28 plasma-soluble ICs using a Luminex 200™ analyzer (Luminex Corporation, Austin, TX, United States). Panel 1 included 14 ICs that play a crucial role in the regulation of T cells, leading to either T cell exhaustion [B and T lymphocyte attenuator (BTLA), cluster of differentiation 80 (CD80), CD152(CTLA4), indoleamine 2,3-dioxygenase (IDO), lymphocyte activation gene-3 (LAG-3), programmed cell death protein 1(PD-1), programmed death-ligand 1 (PD-L1), programmed death-ligand 2 (PD-L2), and T-cell immunoglobulin and mucin-domain containing-3 (TIM-3)] or stimulation [CD27, CD28, CD137(4-1BB), glucocorticoid-induced TNFR-related (GITR), and herpesvirus entry mediator (HVEM)]. Panel 2 included ICs that regulate NK cell activation by the balance of inhibitory [MHC class I chain-related gene A (MICA), MHC class I chain-related gene B (MICB), perforin, human ligand for binding protein 1 (ULBP-1), human ligand for binding protein 3 (ULBP-3), ABD human ligand for binding protein 4 (ULBP-4)] and activating signals [Arginase-1, NT5E(CD73), Tactile(CD96), E-Cadherin, Nectin-2(CD112), PVR(CD155), Siglec-7, and Siglec-9].

Additionally, 26 senescence-associated secretory phenotype (SASP) biomarkers were also evaluated using the Luminex 200™ system (Luminex Corporation, Austin, TX, United States): epidermal growth factor (EGF), eotaxin, chemokine growth-regulated protein alpha (GRO-alpha/KC), granulocyte–macrophage colony-stimulating factor (GM-CSF), interferon-gamma (IFN-gamma), IL-1 beta, IL-1alpha, IL-1RA, IL-2, IL-6, IL-7, IL-8(CXCL10), IL-13, IL-15, IL-18, C-X-C motif chemokine ligand 10 (IP-10(CXCL10)), C–C motif chemokine ligand 2 (MCP-1(CCL2)), C–C motif chemokine ligand 5 (RANTES), stromal cell-derived factor 1-alpha (SDF-1alpha(CXCL12)), fibroblast growth factor 2 (FGF-2), hepatocyte growth factor (HGF), nerve growth factor beta (Beta-NGF), placental growth factor (PIGF-1), skp, cullin, F-box containing complex (SCF), tumor necrosis factor (TNF) alpha, and TNF-beta.

The measured raw fluorescence intensity (FI) values (arbitrary units, a.u.) were used as previously described [59].

### Statistical analysis

For the descriptive study, quantitative variables (clinical and epidemiological variables) were expressed as median (interquartile range, IQR), and categorical variables were shown as absolute count (percentage). Independent groups were compared using the Mann–Whitney U and Chi-square tests for quantitative and categorical variables, respectively. Generalized Linear Models (GLM) with gamma distribution (log-link) were used to estimate differences in the ICs and SASP biomarkers levels between SC and C groups. This test provides the arithmetic mean ratio (AMR), the 95% of confidence interval (95% CI), and its level of significance. Moreover, we adjusted the GLM models for the main available epidemiological and clinical characteristics (age, sex, *IFN*-*λ*_*4*_ genotype (rs12979860), and aspartate transaminase (AST)). These covariates were previously selected by a stepwise method (forward), where at each step, the covariates were considered to enter according to the lowest AKAike information criteria (AIC) for that specific model. Fibrosis-4 index (FIB4) and AST to platelet ratio index (APRI) were not considered covariates in the GLM models because AST was used to calculate these scores. Likewise, alanine transaminase (ALT) was not considered because of its collinearity with AST.

Additionally, differences were estimated between sex for each study group. In this case, GLM models were also adjusted for the same epidemiological and clinical characteristics using a stepwise method (forward). The time from clearance was also included as covariate when analyses were performed by sex within the SC group.

Correlation between significant ICs and SASP proteins was performed using the Spearman correlation test. Those suitable correlations (r > 0.5 or r < -0.5) with a significance value (*p* < 0.05; q-value < 0.10) were considered relevant.

All *p*-values were corrected for multiple testing by using the Benjamini and Hochberg procedure. The significance level was defined as *p*-value < 0.05 (two-tailed) and q-value < 0.10. The statistical analysis was done with R statistical package (R version 4.2.0. R Foundation for Statistical Computing, Vienna, Austria).

### Supplementary Information

**Additional file 1.** Biochemical characteristics of 56 individuals stratified by HCV infection status and sex.

**Additional file 2.** Comparison of plasma immune checkpoints between subjects who spontaneously cleared HCV versus the control group.

**Additional file 3.** Comparison of plasma immune checkpoints between males who spontaneously cleared HCV versus the control group.

**Additional file 4.** Comparison of plasma immune checkpoint proteins between females who spontaneously cleared HCV (SC group) versus controls (C group).

**Additional file 5.** Comparison of senescence-associated secretory phenotype (SASP) proteins between subjects who spontaneously cleared HCV (SC group) versus controls (C group).

**Additional file 6.** Comparison of senescence-associated secretory phenotype (SASP) proteins between males who spontaneously cleared HCV (SC group) versus controls (C group).

**Additional file 7.** Comparison of senescence-associated secretory phenotype (SASP) proteins between females who spontaneously cleared HCV (SC group) versus controls (C group).

**Additional file 8.** Comparison of plasma immune checkpoints between sex in both spontaneous clearance (SC) and control (C) groups: A) SC, B) C.

**Additional file 9.** Comparison of senescence-associated secretory phenotype (SASP) proteins between sex in both spontaneous clearance (SC) and control (C) groups: A) SC, B) C.

**Additional file 10. **Spearman correlation plot between significant immune checkpoint and senescence-associated secretory phenotype (SASP) proteins in males: A) ALL males, B) spontaneous clearance (SC), C) control (C) group.

**Additional file 11. **Spearman correlation plot between significant immune checkpoint and senescence-associated secretory phenotype (SASP) proteins in males: A) ALL males, B) spontaneous clearance (SC), C) control (C) group.

## Data Availability

Not applicable.

## Abbreviations

ALT: Alanine transaminase
AIC: AKAike information criteria
APRI: AST to platelet ratio index
AST: Aspartate transaminase
Beta-NGF: Nerve growth factor beta
BMI: Body mass index
BTLA: B and T lymphocyte attenuator
C: Control
CD: Cluster of differentiation
CNM: Centro Nacional de Microbiología
CVD: Cardiovascular disease
FIB4: Fibrosis-4 index
GGT: Gamma-glutamyl transferase
GITR: Glucocorticoid-induced TNFR-related
GLM: Generalized Linear Models
HCC: Hepatocellular carcinoma
HBV: Hepatitis B virus
HCV: Hepatitis C virus
HVEM: Herpesvirus entry mediator
IC: Immune checkpoint
IDO: Indoleamine 2,3-dioxygenase
IFN-gamma: Interferon-gamma
IL: Interleukin
IP-10: C-X-C motif chemokine ligand 10
irAEs: Immune-related adverse events
IQR: Interquartile range
FGF-2: Fibroblast growth factor 2
FI: Fluorescence intensity
HGF: Hepatocyte growth factor
HOMA IR: Homeostatic model assessment for insulin resistance
LAG-3: Lymphocyte activation gene-3
MAFLD: Metabolic associated fatty liver disease
MCP-1: C-C motif chemokine ligand 2
METS IR: Metabolic score for insulin resistance
MICA: MHC class I chain-related gene A
MICB: MHC class I chain-related gene B
PD-1: Programmed cell death protein 1
PD-L1: Programmed death-ligand 1
PD-L2: Programmed death-ligand 2
PIGF-1: Placental growth factor
TNF: Tumor necrosis factor
TIM-3: T-cell immunoglobulin and mucin-domain containing-3
TyG: Triglyceride glucose index
RANTES: C-C motif chemokine ligand 5
SASP: Senescence-associated secretory phenotype
SC: Spontaneous clearance
SCF: F-box containing complex
SDF-1alpha: Stromal cell-derived factor 1-alpha
ULBP-1: Human ligand for binding protein 1
ULBP-3: Human ligand for binding protein 3
ULBP-4: Human ligand for binding protein 4
VIS: Viral-induced senescence

## References

[1] Spearman CW, Dusheiko GM, Hellard M, Sonderup M (2019). Hepatitis C. Lancet.

[2] Grebely J, Page K, Sacks-Davis R, van der Loeff MS, Rice TM, Bruneau J, Morris MD, Hajarizadeh B, Amin J, Cox AL (2014). The effects of female sex, viral genotype, and IL28B genotype on spontaneous clearance of acute hepatitis C virus infection. Hepatology.

[3] Bulteel N, ParthaSarathy P, Forrest E, Stanley AJ, Innes H, Mills PR, Valerio H, Gunson RN, Aitken C, Morris J (2016). Factors associated with spontaneous clearance of chronic hepatitis C virus infection. J Hepatol.

[4] Page K, Hahn JA, Evans J, Shiboski S, Lum P, Delwart E, Tobler L, Andrews W, Avanesyan L, Cooper S (2009). Acute hepatitis C virus infection in young adult injection drug users: a prospective study of incident infection, resolution, and reinfection. J Infect Dis.

[5] GhamarTalepoor A, Doroudchi M (2022). Immunosenescence in atherosclerosis: a role for chronic viral infections. Front Immunol.

[6] Wykes MN, Lewin SR (2018). Immune checkpoint blockade in infectious diseases. Nat Rev Immunol.

[7] Shoukry NH, Walker CM (2021). T cell responses during HBV and HCV infections: similar but not quite the same?. Curr Opin Virol.

[8] Wang SJ, Dougan SK, Dougan M. Immune mechanisms of toxicity from checkpoint inhibitors. Trends Cancer. 2023;9(7):543–53. 10.1016/j.trecan.2023.04.002 PMC10330206 37117135

[9] Martins F, Sofiya L, Sykiotis GP, Lamine F, Maillard M, Fraga M, Shabafrouz K, Ribi C, Cairoli A, Guex-Crosier Y (2019). Adverse effects of immune-checkpoint inhibitors: epidemiology, management and surveillance. Nat Rev Clin Oncol.

[10] Lian J, Yue Y, Yu W, Zhang Y (2020). Immunosenescence: a key player in cancer development. J Hematol Oncol.

[11] Hernandez-Segura A, Nehme J, Demaria M (2018). Hallmarks of cellular senescence. Trends Cell Biol.

[12] Santoro A, Bientinesi E, Monti D (2021). Immunosenescence and inflammaging in the aging process: age-related diseases or longevity?. Ageing Res Rev.

[13] Fulop T, Larbi A, Dupuis G, Le Page A, Frost EH, Cohen AA, Witkowski JM, Franceschi C (1960). Immunosenescence and inflamm-aging as two sides of the same coin: friends or foes?. Front Immunol.

[14] de Paula HHS, Ferreira ACG, Caetano DG, Delatorre E, Teixeira SLM, Coelho LE, Joao EG, de Andrade MM, Cardoso SW, Grinsztejn B (2018). Reduction of inflammation and T cell activation after 6 months of cART initiation during acute, but not in early chronic HIV-1 infection. Retrovirology.

[15] Osuji FN, Onyenekwe CC, Ahaneku JE, Ukibe NR (2018). The effects of highly active antiretroviral therapy on the serum levels of pro-inflammatory and anti-inflammatory cytokines in HIV infected subjects. J Biomed Sci.

[16] Chan YT, Cheong HC, Tang TF, Rajasuriar R, Cheng KK, Looi CY, Wong WF, Kamarulzaman A. Immune checkpoint molecules and glucose metabolism in hiv-induced T cell exhaustion. Biomedicines. 2022;10(11):0. 10.3390/biomedicines10112809 PMC9687279 36359329

[17] Osuch S, Metzner KJ, Caraballo Cortes K. Reversal of T cell exhaustion in chronic HCV infection. Viruses. 2020;12(8):799. 10.3390/v12080799 PMC7472290 32722372

[18] Oltmanns C, Liu Z, Mischke J, Tauwaldt J, Mekonnen YA, Urbanek-Quaing M, Debarry J, Maasoumy B, Wedemeyer H, Kraft ARM (2023). Reverse inflammaging: long-term effects of HCV cure on biological age. J Hepatol.

[19] Hengst J, Falk CS, Schlaphoff V, Deterding K, Manns MP, Cornberg M, Wedemeyer H (2016). Direct-acting antiviral-induced hepatitis C virus clearance does not completely restore the altered cytokine and chemokine milieu in patients with chronic hepatitis C. J Infect Dis.

[20] Khera T, Du Y, Todt D, Deterding K, Strunz B, Hardtke S, Aregay A, Port K, Hardtke-Wolenski M, Steinmann E (2022). Long-lasting imprint in the soluble inflammatory milieu despite early treatment of acute symptomatic hepatitis C. J Infect Dis.

[21] Hakim MS, Rahmadika N, Jariah ROA (2020). Expressions of inhibitory checkpoint molecules in acute and chronic HBV and HCV infections: implications for therapeutic monitoring and personalized therapy. Rev Med Virol.

[22] Urbani S, Amadei B, Tola D, Massari M, Schivazappa S, Missale G, Ferrari C (2006). PD-1 expression in acute hepatitis C virus (HCV) infection is associated with HCV-specific CD8 exhaustion. J Virol.

[23] Bowen DG, Shoukry NH, Grakoui A, Fuller MJ, Cawthon AG, Dong C, Hasselschwert DL, Brasky KM, Freeman GJ, Seth NP (2008). Variable patterns of programmed death-1 expression on fully functional memory T cells after spontaneous resolution of hepatitis C virus infection. J Virol.

[24] Ackermann C, Smits M, Woost R, Eberhard JM, Peine S, Kummer S, Marget M, Kuntzen T, Kwok WW, Lohse AW (2019). HCV-specific CD4+ T cells of patients with acute and chronic HCV infection display high expression of TIGIT and other co-inhibitory molecules. Sci Rep.

[25] Zoldan K, Ehrlich S, Killmer S, Wild K, Smits M, Russ M, Globig AM, Hofmann M, Thimme R, Boettler T (2021). Th1-biased hepatitis C virus-specific follicular T helper-like cells effectively support B cells after antiviral therapy. Front Immunol.

[26] Smits M, Zoldan K, Ishaque N, Gu Z, Jechow K, Wieland D, Conrad C, Eils R, Fauvelle C, Baumert TF (2020). Follicular T helper cells shape the HCV-specific CD4+ T cell repertoire after virus elimination. J Clin Invest.

[27] Baz-Martinez M, Da Silva-Alvarez S, Rodriguez E, Guerra J, El Motiam A, Vidal A, Garcia-Caballero T, Gonzalez-Barcia M, Sanchez L, Munoz-Fontela C (2016). Cell senescence is an antiviral defense mechanism. Sci Rep.

[28] Wandrer F, Han B, Liebig S, Schlue J, Manns MP, Schulze-Osthoff K, Bantel H (2018). Senescence mirrors the extent of liver fibrosis in chronic hepatitis C virus infection. Aliment Pharmacol Ther.

[29] Tacke F, Trautwein C, Yagmur E, Hellerbrand C, Wiest R, Brenner DA, Schnabl B (2007). Up-regulated eotaxin plasma levels in chronic liver disease patients indicate hepatic inflammation, advanced fibrosis and adverse clinical course. J Gastroenterol Hepatol.

[30] Ferrari SM, Fallahi P, Ruffilli I, Elia G, Ragusa F, Paparo SR, Patrizio A, Mazzi V, Colaci M, Giuggioli D (2019). Immunomodulation of CXCL10 secretion by hepatitis C virus: could CXCL10 be a prognostic marker of chronic hepatitis C?. J Immunol Res.

[31] You CR, Park SH, Jeong SW, Woo HY, Bae SH, Choi JY, Sung YC, Yoon SK (2011). Serum IP-10 levels correlate with the severity of liver histopathology in patients infected with Genotype-1 HCV. Gut Liver.

[32] Lopez Angel CJ, Pham EA, Du H, Vallania F, Fram BJ, Perez K, Nguyen T, Rosenberg-Hasson Y, Ahmed A, Dekker CL, et al. Signatures of immune dysfunction in HIV and HCV infection share features with chronic inflammation in aging and persist after viral reduction or elimination. Proc Natl Acad Sci USA. 2021;118(14):e2022928118. 10.1073/pnas.2022928118 PMC8040665 33811141

[33] Baden R, Rockstroh JK, Buti M (2014). Natural history and management of hepatitis C: does sex play a role?. J Infect Dis.

[34] Badeaux MD, Rolig AS, Agnello G, Enzler D, Kasiewicz MJ, Priddy L, Wiggins JF, Muir A, Sullivan MR, Van Cleef J (2021). Arginase therapy combines effectively with immune checkpoint blockade or agonist anti-OX40 immunotherapy to control tumor growth. Cancer Immunol Res.

[35] Cao W, Sun B, Feitelson MA, Wu T, Tur-Kaspa R, Fan Q (2009). Hepatitis C virus targets over-expression of arginase I in hepatocarcinogenesis. Int J Cancer.

[36] Antonelli A, Ferrari SM, Ruffilli I, Fallahi P (2014). Cytokines and HCV-related autoimmune disorders. Immunol Res.

[37] Giannakoulis VG, Dubovan P, Papoutsi E, Kataki A, Koskinas J. Senescence in HBV-, HCV- and NAFLD-mediated hepatocellular carcinoma and senotherapeutics: current evidence and future perspective. Cancers (Basel). 2021;13(18):4732. 10.3390/cancers13184732 PMC8468315 34572959

[38] Bouman A, Heineman MJ, Faas MM (2005). Sex hormones and the immune response in humans. Hum Reprod Update.

[39] Ng M, Hazrati LN (2022). Evidence of sex differences in cellular senescence. Neurobiol Aging.

[40] Muraro E, Romano R, Fanetti G, Vaccher E, Turturici I, Lupato V, La Torre FB, Polesel J, Fratta E, Giacomarra V (2022). Tissue and circulating PD-L2: moving from health and immune-mediated diseases to head and neck oncology. Crit Rev Oncol Hematol.

[41] Koike K, Takaki A, Yagi T, Iwasaki Y, Yasunaka T, Sadamori H, Shinoura S, Umeda Y, Yoshida R, Sato D (2015). Enhancement of programmed death ligand 2 on hepatitis C virus infected hepatocytes by calcineurin inhibitors. Transplantation.

[42] Horvath S, Levine AJ (2015). HIV-1 infection accelerates age according to the epigenetic clock. J Infect Dis.

[43] Ussher JE, Bilton M, Attwod E, Shadwell J, Richardson R, de Lara C, Mettke E, Kurioka A, Hansen TH, Klenerman P (2014). CD161++ CD8+ T cells, including the MAIT cell subset, are specifically activated by IL-12+IL-18 in a TCR-independent manner. Eur J Immunol.

[44] Dusseaux M, Martin E, Serriari N, Peguillet I, Premel V, Louis D, Milder M, Le Bourhis L, Soudais C, Treiner E (2011). Human MAIT cells are xenobiotic-resistant, tissue-targeted, CD161hi IL-17-secreting T cells. Blood.

[45] Larrubia JR, Moreno-Cubero E, Lokhande MU, Garcia-Garzon S, Lazaro A, Miquel J, Perna C, Sanz-de-Villalobos E (2014). Adaptive immune response during hepatitis C virus infection. World J Gastroenterol.

[46] Carvalho F (2009). How bad is accelerated senescence in consumers of drugs of abuse?. Adicciones.

[47] Friedman H, Pross S, Klein TW (2006). Addictive drugs and their relationship with infectious diseases. FEMS Immunol Med Microbiol.

[48] Kaushik KS, Kapila K, Praharaj AK (2011). Shooting up: the interface of microbial infections and drug abuse. J Med Microbiol.

[49] Pellegrino TC, Dunn KL, Bayer BM (2001). Mechanisms of cocaine-induced decreases in immune cell function. Int Immunopharmacol.

[50] Wang CQ, Li Y, Douglas SD, Wang X, Metzger DS, Zhang T, Ho WZ (2005). Morphine withdrawal enhances hepatitis C virus replicon expression. Am J Pathol.

[51] Ye L, Wang X, Metzger DS, Riedel E, Montaner LJ, Ho W (2010). Upregulation of SOCS-3 and PIAS-3 impairs IL-12-mediated interferon-gamma response in CD56 T cells in HCV-infected heroin users. PLoS ONE.

[52] Ye L, Peng JS, Wang X, Wang YJ, Luo GX, Ho WZ (2008). Methamphetamine enhances hepatitis C virus replication in human hepatocytes. J Viral Hepat.

[53] Feld JJ, Bruneau J, Dore GJ, Ghany MG, Hansen B, Sulkowski M, Thomas DL (2023). Controlled human infection model for hepatitis C virus vaccine development: trial design considerations. Clin Infect Dis.

[54] Liang TJ, Feld JJ, Shoukry NH, Thomas DL (2023). Controlled human infection model for hepatitis C virus vaccine development: is it time to be real?. Clin Infect Dis.

[55] Barnes E, Cooke GS, Lauer GM, Chung RT (2023). Implementation of a controlled human infection model for evaluation of HCV vaccine candidates. Hepatology.

[56] Omland LH, Christensen PB, Krarup H, Jepsen P, Weis N, Sorensen HT, Obel N, Study DC (2011). Mortality among patients with cleared hepatitis C virus infection compared to the general population: a Danish nationwide cohort study. PLoS ONE.

[57] Crespo J, Cuadrado A, Perello C, Cabezas J, Llerena S, Llorca J, Cedillo S, Llop E, Escudero MD, Hernandez Conde M (2020). Epidemiology of hepatitis C virus infection in a country with universal access to direct-acting antiviral agents: data for designing a cost-effective elimination policy in Spain. J Viral Hepat.

[58] Anthony S (1999). Weiss JMJ: sex determination using the polymerase chain reaction. Biochem Educ.

[59] Breen EJ, Polaskova V, Khan A (2015). Bead-based multiplex immuno-assays for cytokines, chemokines, growth factors and other analytes: median fluorescence intensities versus their derived absolute concentration values for statistical analysis. Cytokine.

