# Supplementary material for: Immunological and senescence biomarker profiles in patients after spontaneous clearance of hepatitis C virus: gender implications for long-term health risk
Source: Immun Ageing. 2023 Nov 17;20:62. doi: 10.1186/s12979-023-00387-z (PMC10655350; doi:10.1186/s12979-023-00387-z)
Supplement: Supplementary file 1 — Additional file 1. Biochemical characteristics of 56 individuals stratified by HCV infection status and sex. [file 12979_2023_387_MOESM1_ESM.docx]

**Additional File 1**. Biochemical characteristics of 56 individuals stratified by HCV infection status and sex.

|  | **All** | | | **Male** | | | **Female** | | |
| --- | --- | --- | --- | --- | --- | --- | --- | --- | --- |
|  | SC | C | *p* | SC | C | *p* | SC | C | *p* |
| No. | 32 (57.1%) | 24 (42.9%) |  | 16 | 11 |  | 16 | 13 |  |
| Leukocytes (10^9^/L) | 5.5 (4.8–7.8) | 5.9 (5.3–6.9) | 0.489 | 6.8 (5.7–9.3) | 5.6 (5.3–5.9) | 0.062 | 4.8 (4.5–5.9) | 6.7 (5.6–7.6) | **0.015** |
| Erythrocytes (10^9^/L) | – | 5.0 (4.50–5.4) | – | – | 5.2 (5.1–5.9) | – | – | 4.5 (4.3–4.8) | – |
| Platelets (10^9^/L) | 205.0 (163.0–232.0) | 247.0 (215.0–267.5) | **0.006** | 207.0 (167.5–233.0) | 219.0 (199.0–253.8) | 0.341 | 187.5 (166.0–225.8) | 258.0 (241.0–277.5) | **0.010** |
| Hematocrit | – | 42.7 (38.1–46.4) | – | – | 46.4 (44.2–47.6) | – | – | 41.0 (37.5–42.7) | – |
| Hemoglobin (g/dL) | 13.8 (12.9–14.7) | 14.3 (12.8–15.6) | 0.286 | 14.8 (14.5–15.3) | 15.6 (15.2–16.1) | 0.072 | 12.9 (12.5–13.4) | 13.5 (12.7–14.3) | 0.134 |
| Albumin (g/dL) | 4.3 (4.2–4.5) | – | – | 4.4 (4.3–4.6) | – | – | 4.3 (4.1–4.3) | – | – |
| Homocysteine (µmol/L) | 12.0 (10.7–15.6) | – | – | 12.5 (11.2–14.7) | – | – | 10.9 (10.0–20.2) | – | – |
| Alkaline phosphatase (U/L) | 73.5 (58.8–82.5) | – | – | 72.5 (57.5–81.3) | – | – | 73.5 (61.3–85.5) | – | – |
| Total Bilirubin (mg/dL) | 0.5 (0.4–0.7) | 0.4 (0.3–0.7) | 0.266 | 0.5 (0.4–0.7) | 0.5 (0.4–0.7) | 0.441 | 0.5 (0.3–0.6) | 0.4 (0.3–0.6) | 0.629 |
| Glucose (mg/dL) | 88.0 (80.8–99.5) | 91.0 (80.0–93.5) | 0.891 | 88.0 (84.8–95.3) | 91.5 (88.8–92.8) | 0.853 | 89.0 (70.8–107.3) | 88.0 (79.0–95.0) | 0.895 |
| TC (mg/dL) | 192.0 (174.0–207.0) | 208.0 (189.0–224.5) | 0.085 | 194.0 (174.0–200.0) | 205.5 (188.0–220.5) | 0.157 | 190.0 (176.8–207.5) | 210.0 (194.0–226.0) | 0.228 |
| HDL (mg/dL) | 55.0 (46.5–64.5) | 67.0 (51.5–80.0) | 0.052 | 46.0 (39.0–54.0) | 56.5 (46.8–63.3) | 0.191 | 60.5 (54.3–70.0) | 77.0 (67.0–84.0) | 0.055 |
| LDL (mg/dL) | 107.0 (89.5–115.5) | – | – | 107.0 (102.0–122.0) | – | – | 108.5 (89.3–114.5) | – | – |
| LDL/HDL | 1.9 (1.5–2.3) | – | – | 2.2 (2.1–2.4) | – | – | 1.7 (1.5–2.0) | – | – |
| TC/HDL | 3.3 (3.0–3.8) | 3.1 (2.6–3.8) | 0.407 | 3.7 (3.4–4.1) | 3.8 (3.3–4.3) | 0.999 | 3.1 (2.9–3.4) | 2.7 (2.6–3.1) | 0.239 |
| TG (mg/dL) | 91.0 (72.0–127.5) | 87.0 (71.0–132.0) | 0.860 | 87.0 (69.0–127.5) | 132.0 (95.3–156.5) | 0.415 | 97.5 (77.3–129.3) | 74.0 (67.0–89.0) | 0.077 |
| TG/HDL | 1.5 (1.3–2.2) | 1.4 (0.9–2.4) | 0.491 | 1.5 (1.5–1.6) | 2.3 (1.7–3.3) | 0.254 | 1.6 (1.2–2.2) | 1.0 (0.8–1.4) | **0.030** |
| AIP | 0.2 (0.1–0.3) | 0.2 (0.0–0.4) | 0.491 | 0.2 (0.2–0.2) | 0.4 (0.2–0.5) | 0.254 | 0.2 (0.1–0.4) | 0.1 (0.0–0.4) | **0.030** |
| LCI/10^3^ | 30.8 (25.2–35.0) | – | – | 30.8 (26.0–32.2) | – | – | 31.3 (25.2–48.0) | – | – |
| Urea (mg/dL) | – | 33.0 (29.0–38.0) | – | – | 38.0 (33.8–41.0) | – | – | 32.0 (26.0–34.0) | – |
| Creatinine (mg/dL) | – | 0.7 (0.7–1.0) | – | – | 1.0 (0.9–1.0) | – | – | 0.7 (0.6–0.7) | – |
| Uric acid (mg/dL) | – | 4.9 (4.1–5.9) | – | – | 5.9 (5.4–7.0) | – | – | 4.2 (3.4–4.9) | – |
| Ferritin (ng/mL) | – | 89.0 (36.2–199.9) | – | – | 172.2 (119.3–218.6) | – | – | 42.9 (20.2–89.0) | – |
| Sodium (mEq/L) | – | 142.8 (141.1–143.4) | – | – | 142.5 (141.2–143.8) | – | – | 142.8 (141.0–143.1) | – |
| Potassium (mEq/L) | – | 4.6 (4.3–4.7) | – | – | 4.7 (4.4–4.9) | – | – | 4.5 (4.2–4.7) | – |
| Calcium (mg/dL) | – | 9.3 (9.2–9.7) | – | – | 9.4 (9.2–9.5) | – | – | 9.4 (9.2–9.8) | – |
| Phosphorus (mg/dL) | – | 3.1 (2.6–3.4) | – | – | 2.8 (2.6–3.2) | – | – | 3.2 (3.0–3.6) | – |
| Iron (µg/dL) | – | 84.0 (62.0–119.0) | – | – | 104.0 (85.0–120.3) | – | – | 80.0 (61.0–89.0) | – |

**Statistics**: The values are expressed as the absolute number (percentage) and median (interquartile range). P-values were calculated by the Chi-square test and the Mann-Whitney U test.

**Abbreviations**: AIP, atherogenic index of plasma; C; control; HCV, hepatitis C virus; HDL, high-density lipoprotein; LCI, lipoprotein combine index; LDL, low-density lipoprotein; SC, spontaneous clearance; TC, total cholesterol; TG, triglycerides.
