# Supplementary material for: Immunological and senescence biomarker profiles in patients after spontaneous clearance of hepatitis C virus: gender implications for long-term health risk
Source: Immun Ageing. 2023 Nov 17;20:62. doi: 10.1186/s12979-023-00387-z (PMC10655350; doi:10.1186/s12979-023-00387-z)
Supplement: Supplementary file 2 — Additional file 2. Comparison of plasma immune checkpoints between subjects who spontaneously cleared HCV versus the control group. [file 12979_2023_387_MOESM2_ESM.docx]

**Additional File 2.** Comparison of plasma immune checkpoint proteins between subjects who spontaneously cleared HCV (SC group) versus controls (C group).

|  | **Un-adjusted** | | | **Adjusted** | | |
| --- | --- | --- | --- | --- | --- | --- |
| **Marker** | **AMR (95%CI)** | ***p*-value** | ***q*-value** | **aAMR (95%CI)** | ***p*-value** | ***q*-value** |
| BTLA | 1.37 (1.16–1.63) | **<0.001** | **0.004** | 1.35 (1.14–1.60) | **<0.001** | **0.004** |
| CD137(4-1BB) | 1.46 (1.21–1.77) | **<0.001** | **0.003** | 1.46 (1.22–1.75) | **<0.001** | **0.002** |
| CD152(CTLA4) | 1.39 (1.02–1.88) | **0.041** | **0.083** | 1.37 (1.00–1.88) | 0.054 | 0.108 |
| CD27 | 1.38 (1.05–1.81) | **0.024** | **0.057** | 1.44 (1.11–1.87) | **0.008** | **0.022** |
| CD28 | 1.38 (1.04–1.82) | **0.027** | **0.059** | 1.39 (1.06–1.84) | **0.023** | **0.048** |
| CD80 | 1.46 (1.15–1.85) | **0.003** | **0.011** | 1.47 (1.17–1.86) | **0.002** | **0.007** |
| GITR | 1.54 (1.22–1.94) | **<0.001** | **0.004** | 1.55 (1.24–1.96) | **<0.001** | **0.003** |
| HVEM | 1.33 (1.11–1.59) | **0.003** | **0.011** | 1.38 (1.16–1.64) | **<0.001** | **0.003** |
| IDO | 1.46 (1.17–1.83) | **0.002** | **0.009** | 1.47 (1.17–1.84) | **0.002** | **0.006** |
| LAG-3 | 1.46 (1.14–1.87) | **0.004** | **0.011** | 1.44 (1.12–1.86) | **0.007** | **0.019** |
| PD-1 | 1.40 (1.13–1.75) | **0.004** | **0.011** | 1.46 (1.19–1.81) | **<0.001** | **0.004** |
| PD-L1 | 1.33 (1.06–1.65) | **0.015** | **0.038** | 1.36 (1.08–1.70) | **0.010** | **0.024** |
| PD-L2 | 1.84 (1.46–1.33) | **<0.001** | **<0.001** | 1.82 (1.49–1.23) | **<0.001** | **<0.001** |
| TIM-3 | 1.42 (1.13–1.78) | **0.004** | **0.011** | 1.49 (1.20–1.84) | **<0.001** | **0.003** |
| Arginase | 0.97 (0.82–1.15) | 0.724 | 0.751 | 0.96 (0.81–1.15) | 0.690 | 0.743 |
| E-Cadherin | 1.31 (0.91–1.87) | 0.147 | 0.229 | 1.23 (0.87–1.73) | 0.251 | 0.351 |
| MICA | 1.11 (0.89–1.39) | 0.355 | 0,452 | 1.03 (0.82–1.29) | 0.773 | 0.801 |
| MICB | 1.10 (0.89–1.34) | 0.384 | 0,461 | 1.11 (0.91–1.35) | 0.324 | 0.413 |
| NT5E(CD73) | 1.15 (0.92–1.44) | 0.220 | 0,307 | 1.13 (0.91–1.41) | 0.282 | 0.376 |
| Nectin-2(CD112) | 1.28 (0.93–1.76) | 0.131 | 0.215 | 1.25 (0.90–1.73) | 0.183 | 0.285 |
| PVR(CD155) | 1.13 (0.89–1.44) | 0.304 | 0.405 | 1.18 (0.94–1.48) | 0.159 | 0.262 |
| Perforin | 1.33 (0.96–1.83) | 0.088 | 0.164 | 1.31 (0.95–1.81) | 0.111 | 0.195 |
| Siglec-7 | 1.22 (0.95–1.55) | 0.121 | 0.212 | 1.15 (1.92–1.44) | 0.217 | 0.320 |
| Siglec-9 | 1.22 (0.92–1.62) | 0.174 | 0.256 | 1.30 (1.00–1.71) | 0.060 | 0.111 |
| Tactile(CD96) | 0.88 (0.66–1.18) | 0.395 | 0.461 | 0.91 (0.68–1.21) | 0.509 | 0.594 |
| ULBP-1 | 0.92 (0.67–1.26) | 0.604 | 0.651 | 0.89 (0.64–1.24) | 0.490 | 0.594 |
| ULBP-3 | 0.98 (0.77–1.24) | 0.860 | 0.860 | 0.97 (0.76–1.24) | 0.817 | 0.817 |
| ULBP-4 | 1.04 (0.91–1.18) | 0.575 | 0.643 | 1.03 (0.90–1.19) | 0.626 | 0.701 |

**Statistics:** Data were calculated by Generalized Linear Models (GLM) with a gamma distribution (log-link). Multivariable models were adjusted by age, sex, *IFN-λ_3_* genotype, and AST, previously selected by a stepwise method (forward) (see **Results Section**). The q-values represent p-values corrected for multiple testing using the False Discovery Rate (FDR). Significant differences are shown in bold.

**Abbreviations**: AMR, arithmetic mean ratio; aAMR, adjusted AMR; 95%CI, 95% of confidence interval; p, level of significance; q, corrected level of significance; BTLA, B and T lymphocyte attenuator; CD, cluster of differentiation; GITR, glucocorticoid-induced TNFR-related; HVEM, herpesvirus entry mediator; IDO, indoleamine 2,3-dioxygenase; LAG-3, lymphocyte activation gene-3; PD-1, programmed cell death protein 1; PD-L1, programmed death-ligand 1; PD-L2, programmed death-ligand 2; TIM-3, T-cell immunoglobulin and mucin-domain containing-3; MICA, MHC class I chain-related gene A; MICB, MHC class I chain-related gene B; NT5E, ecto-5′-nucleotidase; PVR, poliovirus receptor; Siglec, sialic acid-binding immunoglobulin-type lectin; ULBP, human ligand for binding protein.
