# Supplementary material for: Immunological and senescence biomarker profiles in patients after spontaneous clearance of hepatitis C virus: gender implications for long-term health risk
Source: Immun Ageing. 2023 Nov 17;20:62. doi: 10.1186/s12979-023-00387-z (PMC10655350; doi:10.1186/s12979-023-00387-z)
Supplement: Supplementary file 3 — Additional file 3. Comparison of plasma immune checkpoints between males who spontaneously cleared HCV versus the control group. [file 12979_2023_387_MOESM3_ESM.docx]

**Additional File 3.** Comparison of plasma immune checkpoint proteins between males who spontaneously cleared HCV (SC group) versus controls (C group).

|  | **Un-adjusted** | | | **Adjusted** | | |
| --- | --- | --- | --- | --- | --- | --- |
| **Marker** | **AMR (95%CI)** | ***p*-value** | ***q*-value** | **aAMR (95%CI)** | ***p*-value** | ***q*-value** |
| BTLA | 1.48 (1.19–1.85) | **0.002** | **0.007** | 1.49 (1.18–1.88) | **0.003** | **0.014** |
| CD137(4-1BB) | 1.56 (1.19–2.03) | **0.003** | **0.010** | 1.53 (1.17–2.02) | **0.005** | **0.017** |
| CD152(CTLA4) | 1.20 (0.79–1.83) | 0.392 | 0.478 | 1.19 (0.77–1.83) | 0.441 | 0.537 |
| CD27 | 1.54 (0.10–2.33) | 0.055 | 0.091 | 1.53 (0.99–2.37) | 0.070 | 0.122 |
| CD28 | 1.45 (0.94–2.22) | 0.105 | 0.155 | 1.40 (0.90–2.20) | 0.149 | 0.209 |
| CD80 | 1.50 (1.15–1.96) | **0.006** | **0.016** | 1.48 (1.12–1.96) | **0.011** | **0.031** |
| GITR | 1.73 (1.26–2.38) | **0.002** | **0.008** | 1.73 (1.24–2.41) | **0.004** | **0.014** |
| HVEM | 1.36 (1.09–1.69) | **0.011** | **0.026** | 1.38 (1.12–1.69) | **0.006** | **0.018** |
| IDO | 1.66 (1.27–2.16) | **0.001** | **0.007** | 1.65 (1.25–2.16) | **0.002** | **0.014** |
| LAG-3 | 1.66 (1.25–2.21) | **0.002** | **0.007** | 1.64 (1.22–2.20) | **0.003** | **0.014** |
| PD-1 | 1.57 (1.25–1.98) | **<0.001** | **0.007** | 1.60 (1.26–2.04) | **0.001** | **0.014** |
| PD-L1 | 1.50 (1.11–2.02) | **0.012** | **0.026** | 1.55 (1.13–2.12) | **0.013** | **0.032** |
| PD-L2 | 1.56 (1.22–1.99) | **0.001** | **0.007** | 1.59 (1.22–2.06) | **0.002** | **0.014** |
| TIM-3 | 1.63 (1.26–2.10) | **0.001** | **0.007** | 1.40 (1.07–1.83) | **0.025** | **0.059** |
| Arginase | 1.22 (1.02–1.46) | **0.038** | **0.071** | 1.23 (1.02–1.50) | **0.045** | **0.097** |
| E-Cadherin | 1.90 (1.24–2.91) | **0.006** | **0.016** | 1.56 (0.98–2.48) | 0.075 | 0.124 |
| MICA | 1.40 (1.05–1.87) | **0.032** | **0.063** | 1.37 (1.00–1.87) | 0.059 | 0.118 |
| MICB | 1.28 (1.01–1.62) | 0.052 | 0.091 | 1.28 (1.00–1.64) | 0.066 | 0.122 |
| NT5E(CD73) | 1.09 (0.81–1.46) | 0.572 | 0.618 | 1.06 (0.78–1.45) | 0.702 | 0.760 |
| Nectin-2(CD112) | 1.12 (0.75–1.67) | 0.574 | 0.618 | 1.08 (0.72–1.63) | 0.706 | 0.760 |
| PVR(CD155) | 1.31 (0.93–1.84) | 0.133 | 0.187 | 1.33 (0.95–1.87) | 0.113 | 0.166 |
| Perforin | 1.26 (0.91–1.96) | 0.318 | 0.412 | 1.32 (0.84–2.07) | 0.243 | 0.325 |
| Siglec-7 | 1.18 (0.85–1.65) | 0.324 | 0.412 | 1.15 (0.84–1.57) | 0.389 | 0.495 |
| Siglec-9 | 1.87 (1.31–2.66) | **0.002** | **0.008** | 1.87 (1.29–2.70) | **0.003** | **0.014** |
| Tactile(CD96) | 1.13 (0.76–1.68) | 0.552 | 0.618 | 1.14 (0.75–1.73) | 0.549 | 0.640 |
| ULBP-1 | 0.98 (0.62–1.56) | 0.947 | 0.947 | 1.05 (0.68–1.63) | 0.817 | 0.845 |
| ULBP-3 | 1.33 (1.00–1.78) | 0.063 | 0.098 | 1.30 (0.96–1.76) | 0.108 | 0.166 |
| ULBP-4 | 1.02 (0.84–1.15) | 0.804 | 0.834 | 1.02 (0.83–1.27) | 0.845 | 0.845 |

**Statistics:** Data were calculated by Generalized Linear Models (GLM) with a gamma distribution (log-link). Multivariable models were adjusted by age, IL28 genotype, and AST, previously selected by a stepwise method (forward) (see **Results Section**). The q-values represent p-values corrected for multiple testing using the False Discovery Rate (FDR). Significant differences are shown in bold.

**Abbreviations**: AMR, arithmetic mean ratio; aAMR, adjusted AMR; 95%CI, 95% of confidence interval; p, level of significance; q, corrected level of significance; BTLA, B and T lymphocyte attenuator; CD, cluster of differentiation; GITR, glucocorticoid-induced TNFR-related; HVEM, herpesvirus entry mediator; IDO, indoleamine 2,3-dioxygenase; LAG-3, lymphocyte activation gene-3; PD-1, programmed cell death protein 1; PD-L1, programmed death-ligand 1; PD-L2, programmed death-ligand 2; TIM-3, T-cell immunoglobulin and mucin-domain containing-3; MICA, MHC class I chain-related gene A; MICB, MHC class I chain-related gene B; NT5E, ecto-5′-nucleotidase; PVR, poliovirus receptor; Siglec, sialic acid-binding immunoglobulin-type lectin; ULBP, human ligand for binding protein.
