# Supplementary material for: Immunological and senescence biomarker profiles in patients after spontaneous clearance of hepatitis C virus: gender implications for long-term health risk
Source: Immun Ageing. 2023 Nov 17;20:62. doi: 10.1186/s12979-023-00387-z (PMC10655350; doi:10.1186/s12979-023-00387-z)
Supplement: Supplementary file 4 — Additional file 4. Comparison of plasma immune checkpoint proteins between females who spontaneously cleared HCV (SC group) versus controls (C group). [file 12979_2023_387_MOESM4_ESM.docx]

**Additional File 4.** Comparison of plasma immune checkpoint proteins between females who spontaneously cleared HCV (SC group) versus controls (C group).

|  | **Un-adjusted** | | | **Adjusted** | | |
| --- | --- | --- | --- | --- | --- | --- |
| **Marker** | **AMR (95%CI)** | ***p*-value** | ***q*-value** | **aAMR (95%CI)** | ***p*-value** | ***q*-value** |
| BTLA | 1.31 (1.02–1.67) | **0.041** | 0.263 | 1.24 (0.97–1.57) | 0.098 | 0.211 |
| CD137(4-1BB) | 1.40 (1.07–1.83) | **0.020** | 0.263 | 1.38 (1.08–1.76) | **0.016** | 0.121 |
| CD152(CTLA4) | 1.56 (1.02–2.39) | **0.048** | 0.263 | 1.56 (1.00–2.42) | 0.058 | 0.163 |
| CD27 | 1.30 (0.92–1.83) | 0.150 | 0.263 | 1.27 (0.85–1.90) | 0.254 | 0.374 |
| CD28 | 1.35 (0.94–1.94) | 0.115 | 0.263 | 1.24 (0.87–1.78) | 0.241 | 0.374 |
| CD80 | 1.44 (1.01–2.07) | 0.055 | 0.263 | 1.46 (1.01–2.11) | 0.054 | 0.163 |
| GITR | 1.41 (1.00–1.98) | 0.059 | 0.263 | 1.53 (1.11–2.12) | **0.017** | 0.121 |
| HVEM | 1.31 (0.99–1.73) | 0.071 | 0.263 | 1.44 (1.08–1.91) | **0.019** | 0.121 |
| IDO | 1.34 (0.96–1.88) | 0.101 | 0.263 | 1.23 (0.89–1.71) | 0.228 | 0.374 |
| LAG-3 | 1.33 (0.92–1.93) | 0.139 | 0.263 | 1.46 (1.01–2.09) | 0.053 | 0.163 |
| PD-1 | 1.32 (0.95–1.84) | 0.110 | 0.263 | 1.35 (0.97–1.89) | 0.085 | 0.199 |
| PD-L1 | 1.20 (0.87–1.65) | 0.287 | 0.383 | 1.24 (0.90–1.70) | 0.195 | 0.374 |
| PD-L2 | 2.10 (1.58–2.79) | **<0.001** | **0.001** | 2.19 (1.65–2.91) | **<0.001** | **<0.001** |
| TIM-3 | 1.32 (0.97–1.8*0*) | 0.083 | 0.263 | 1.21 (0.88–1.66) | 0.250 | 0.374 |
| Arginase | 0.79 (0.61–1.02) | 0.086 | 0.263 | 0.72 (0.55–0.94) | **0.022** | 0.121 |
| E-Cadherin | 1.12 (0.70–1.79) | 0.635 | 0.729 | 0.95 (0.58–1.57) | 0.857 | 0.857 |
| MICA | 0.93 (0.67–1.29) | 0.651 | 0.729 | 0.86 (0.63–1.16) | 0.336 | 0.449 |
| MICB | 1.00 (0.75–1.33) | 0.997 | 0.997 | 1.06 (0.80–1.41) | 0.701 | 0.727 |
| NT5E(CD73) | 1.22 (0.90–1.65) | 0.212 | 0.297 | 1.19 (0.87–1.63) | 0.283 | 0.397 |
| Nectin-2(CD112) | 1.43 (0.92–2.24) | 0.128 | 0.263 | 1.53 (0.96–2.42) | 0.083 | 0.199 |
| PVR(CD155) | 1.06 (0.78–1.43) | 0.719 | 0.774 | 0.94 (0.68–1.29) | 0.685 | 0.727 |
| Perforin | 1.40 (0.89–2.21) | 0.154 | 0.263 | 1.17 (0.73–1.89) | 0.519 | 0.609 |
| Siglec-7 | 1.26 (0.92–1.73) | 0.160 | 0.263 | 1.22 (0.88–1.70) | 0.236 | 0.374 |
| Siglec-9 | 0.98 (0.68–1.41) | 0.918 | 0.952 | 0.86 (0.58–1.26) | 0.448 | 0.570 |
| Tactile(CD96) | 0.76 (0.50–1.15) | 0.198 | 0.292 | 0.64 (0.41–0.99) | 0.058 | 0.163 |
| ULBP-1 | 0.88 (0.56–1.36) | 0.560 | 0.681 | 0.86 (0.54–1.36) | 0.522 | 0.609 |
| ULBP-3 | 0.79 (0.56–1.11) | 0.186 | 0.289 | 0.70 (0.50–0.96) | **0.038** | 0.163 |
| ULBP-4 | 1.06 (0.89–1.25) | 0.546 | 0.681 | 1.06 (0.88–1.26) | 0.556 | 0.622 |

**Statistics:** Data were calculated by Generalized Linear Models (GLM) with a gamma distribution (log-link). Multivariable models were adjusted by age, IL28 genotype, and AST, previously selected by a stepwise method (forward) (see **Results Section**). The q-values represent p-values corrected for multiple testing using the False Discovery Rate (FDR). Significant differences are shown in bold.

**Abbreviations**: AMR, arithmetic mean ratio; aAMR, adjusted AMR; 95%CI, 95% of confidence interval; p, level of significance; q, corrected level of significance; BTLA, B and T lymphocyte attenuator; CD, cluster of differentiation; GITR, glucocorticoid-induced TNFR-related; HVEM, herpesvirus entry mediator; IDO, indoleamine 2,3-dioxygenase; LAG-3, lymphocyte activation gene-3; PD-1, programmed cell death protein 1; PD-L1, programmed death-ligand 1; PD-L2, programmed death-ligand 2; TIM-3, T-cell immunoglobulin and mucin-domain containing-3; MICA, MHC class I chain-related gene A; MICB, MHC class I chain-related gene B; NT5E, ecto-5′-nucleotidase; PVR, poliovirus receptor; Siglec, sialic acid-binding immunoglobulin-type lectin; ULBP, human ligand for binding protein.
