# Supplementary material for: Immunological and senescence biomarker profiles in patients after spontaneous clearance of hepatitis C virus: gender implications for long-term health risk
Source: Immun Ageing. 2023 Nov 17;20:62. doi: 10.1186/s12979-023-00387-z (PMC10655350; doi:10.1186/s12979-023-00387-z)
Supplement: Supplementary file 5 — Additional file 5. Comparison of senescence-associated secretory phenotype (SASP) proteins between subjects who spontaneously cleared HCV (SC group) versus controls (C group). [file 12979_2023_387_MOESM5_ESM.docx]

**Additional File 5.** Comparison of senescence-associated secretory phenotype (SASP) proteins between subjects who spontaneously cleared HCV (SC group) versus controls (C group).

|  | **Un-adjusted** | | | **Adjusted** | | |
| --- | --- | --- | --- | --- | --- | --- |
| **Marker** | **AMR (95%CI)** | ***p*-value** | ***q*-value** | **aAMR (95%CI)** | ***p*-value** | ***q*-value** |
| EGF | 1.26 (1.03–1.54) | **0.026** | **0.084** | 1.29 (1.05–1.58) | **0.019** | **0.063** |
| Eotaxin | 1.47 (1.03–2.10) | **0.037** | **0.100** | 1.58 (1.07–2.32) | **0.025** | **0.063** |
| GRO-alpha/KC | 1.10 (0.96–1.27) | 0.187 | 0.243 | 1.12 (0.97–1.30) | 0.139 | 0.201 |
| GM-CSF | 1.19 (0.97–1.46) | 0.103 | 0.168 | 1.19 (0.97–1.48) | 0.105 | 0.160 |
| IFN-gamma | 1.13 (0.97–1.31) | 0.113 | 0.173 | 1.11 (0.95–1.30) | 0.176 | 0.235 |
| IL-1beta | 1.22 (1.01–1.46) | **0.042** | **0.100** | 1.21 (1.00–1.45) | 0.051 | 0.095 |
| IL-1alpha | 1.18 (0.99–1.41) | 0.065 | 0.121 | 1.22 (1.01–1.46) | **0.038** | **0.081** |
| IL-1RA | 1.33 (1.12–1.57) | **0.002** | **0.024** | 1.29 (1.08–1.53) | **0.006** | **0.060** |
| IL-2 | 1.19 (0.93–1.53) | 0.182 | 0.243 | 1.19 (0.92–1.54) | 0.185 | 0.235 |
| IL-6 | 1.08 (0.89–1.33) | 0.438 | 0.495 | 1.09 (0.89–1.34) | 0.406 | 0.459 |
| IL-7 | 1.15 (0.97–1.38) | 0.120 | 0.174 | 1.17 (0.98–1.41) | 0.096 | 0.155 |
| IL-8 | 1.16 (1.00–1.33) | **0.050** | 0.108 | 1.17 (1.01–1.36) | **0.041** | **0.081** |
| IL-13 | 1.24 (1.04–1.48) | **0.022** | **0.081** | 1.24 (1.03–1.49) | **0.025** | **0.063** |
| IL-15 | 1.03 (0.87–1.23) | 0.736 | 0.797 | 1.00 (0.83–1.20) | 0.986 | 0.986 |
| IL-18 | 1.46 (1.12–1.91) | **0.008** | 0.0476 | 1.45 (1.09–1.91) | **0.013** | **0.060** |
| IP-10 | 1.60 (1.12–2.29) | **0.013** | **0.055** | 1.50 (1.06–2.13) | **0.027** | **0.063** |
| MCP-1 | 1.23 (0.87–1.74) | 0.245 | 0.303 | 1.30 (0.88–1.92) | 0.189 | 0.235 |
| RANTES | 1.02 (0.81–1.28) | 0.868 | 0.902 | 0.92 (0.73–1.17) | 0.495 | 0.536 |
| SDF-1alpha | 1.27 (1.07–1.50) | **0.007** | **0.048** | 1.24 (1.04–1.46) | **0.016** | **0.060** |
| FGF-2 | 1.13 (0.98–1.31) | 0.103 | 0.168 | 1.15 (0.98–1.34) | 0.085 | 0.147 |
| HGF | 1.28 (1.07–1.52) | **0.009** | **0.048** | 1.29 (1.07–1.54) | **0.009** | **0.060** |
| Beta-NGF | 1.11 (1.00–1.24) | 0.054 | 0.108 | 1.14 (1.03–1.27) | **0.016** | **0.060** |
| PLGF-1 | 1.28 (1.02–1.62) | **0.038** | **0.100** | 1.40 (1.10–1.80) | **0.010** | **0.060** |
| SCF | 1.33 (1.13–1.57) | **0.001** | **0.024** | 1.31 (1.11–1.55) | **0.002** | **0.060** |
| TNF-alpha | 1.10 (0.94–1.29) | 0.257 | 0.303 | 1.10 (0.93–1.30) | 0.285 | 0.337 |
| TNF-beta | 0.99 (0.80–1.22) | 0.920 | 0.920 | 1.04 (0.84–1.28) | 0.746 | 0.776 |

**Statistics:** Data were calculated by Generalized Linear Models (GLM) with a gamma distribution (log-link). Multivariable models were adjusted by age, sex, IL28 genotype, and AST, previously selected by a stepwise method (forward) (see **Results Section**). The q-values represent p-values corrected for multiple testing using the False Discovery Rate (FDR). Significant differences are shown in bold.

**Abbreviations**: AMR, arithmetic mean ratio; aAMR, adjusted AMR; 95%CI, 95% of confidence interval; p, level of significance; q, corrected level of significance; EGF, epidermal growth factor; GRO-alpha/KC, chemokine growth-regulated protein alpha; GM-CSF, granulocyte macrophage colony-stimulating factor; IFN, interferon; IL, interleukin; MCP-1, C-C motif chemokine ligand 2; RANTES, C-C motif chemokine ligand 5; SDF-1alpha, stromal cell-derived factor 1alpha; FGF-2, fibroblast growth factor 2; HGF, hepatocyte growth factor; Beta-NGF, nerve growth factor β; PLGF-1, placental growth factor; SCF, skp, cullin, F-box containing complex; TNF, tumoral necrosis factor.
