# Supplementary material for: Immunological and senescence biomarker profiles in patients after spontaneous clearance of hepatitis C virus: gender implications for long-term health risk
Source: Immun Ageing. 2023 Nov 17;20:62. doi: 10.1186/s12979-023-00387-z (PMC10655350; doi:10.1186/s12979-023-00387-z)
Supplement: Supplementary file 6 — Additional file 6. Comparison of senescence-associated secretory phenotype (SASP) proteins between males who spontaneously cleared HCV (SC group) versus controls (C group). [file 12979_2023_387_MOESM6_ESM.docx]

**Additional File 6.** Comparison of senescence-associated secretory phenotype (SASP) proteins between males who spontaneously cleared HCV (SC group) versus controls (C group).

|  | **Un-adjusted** | | | **Adjusted** | | |
| --- | --- | --- | --- | --- | --- | --- |
| **Marker** | **AMR (95%CI)** | ***p*-value** | ***q*-value** | **aAMR (95%CI)** | ***p*-value** | ***q*-value** |
| EGF | 1.20 (0.94–1.53) | 0.154 | 0.285 | 1.20 (0.96–1.51) | 0.119 | 0.228 |
| Eotaxin | 1.65 (0.97–2.83) | 0.078 | 0.170 | 1.50 (0.85–2.66) | 0.175 | 0.284 |
| Gro-alpha/KC | 1.02 (0.85–1.23) | 0.802 | 0.864 | 1.06 (0.89–1.27) | 0.504 | 0.570 |
| GM-CSF | 1.31 (0.99–1.75) | 0.074 | 0.170 | 1.32 (1.03–1.69) | **0.038** | 0.113 |
| IFN-gamma | 1.29 (1.06–1.57) | **0.017** | **0.089** | 1.33 (1.08–1.64) | **0.012** | **0.087** |
| IL-1beta | 1.45 (1.10–1.91) | **0.014** | **0.089** | 1.42 (1.06–1.91) | **0.028** | 0.113 |
| IL-1alpha | 1.13 (0.87–1.46) | 0.362 | 0.523 | 1.15 (0.88–1.52) | 0.314 | 0.463 |
| IL-1RA | 1.34 (1.05–1.70) | **0.026** | **0.089** | 1.31 (1.02–1.68) | **0.048** | 0.114 |
| IL-2 | 1.43 (1.05–1.95) | **0.034** | **0.089** | 1.39 (1.02–1.87) | **0.044** | 0.114 |
| IL-6 | 1.20 (0.89–1.61) | 0.235 | 0.399 | 1.17 (0.86–1.60) | 0.321 | 0.463 |
| IL-7 | 1.28 (1.03–1.60) | **0.033** | **0.089** | 1.30 (1.02–1.64) | **0.042** | 0.114 |
| IL-8 | 1.12 (0.93–1.34) | 0.246 | 0.399 | 1.16 (0.97–1.39) | 0.123 | 0.228 |
| IL-13 | 1.34 (1.04–1.71) | **0.031** | **0.089** | 1.30 (1.00–1.69) | 0.060 | 0.130 |
| IL-15 | 1.12 (0.90–1.38) | 0.323 | 0.493 | 1.09 (0.84–1.38) | 0.449 | 0.546 |
| IL-18 | 1.84 (1.36–2.48) | **<0.001** | **0.009** | 1.85 (1.34–2.55) | **0.001** | **0.028** |
| IP-10 | 2.15 (1.45–3.19) | **0.001** | **0.009** | 1.87 (1.18–2.94) | **0.013** | **0.087** |
| MCP-1 | 1.06 (0.62–1.81) | 0.845 | 0.864 | 1.22 (0.74–2.03) | 0.449 | 0.546 |
| RANTES | 0.97 (0.69–1.36) | 0.864 | 0.864 | 1.03 (0.70–1.52) | 0.871 | 0.871 |
| SDF-1alpha | 1.07 (0.85–1.36) | 0.557 | 0.644 | 1.04 (0.84–1.30) | 0.702 | 0.761 |
| FGF-2 | 1.08 (0.89–1.31) | 0.447 | 0.581 | 1.08 (0.88–1.32) | 0.462 | 0.546 |
| HGF | 1.38 (1.06–1.80) | **0.026** | **0.089** | 1.36 (1.02–1.83) | **0.047** | 0.114 |
| BNGF | 1.04 (0.93–1.18) | 0.484 | 0.599 | 1.12 (1.01–1.24) | **0.044** | 0.114 |
| PIGF-1 | 1.14 (0.84–1.54) | 0.398 | 0.545 | 1.14 (0.82–1.58) | 0.440 | 0.546 |
| SCF | 1.41 (1.18–1.69) | 0**.001** | **0.009** | 1.38 (1.14–1.68) | **0.003** | **0.044** |
| TNF-alpha | 1.17 (0.95–1.43) | 0.144 | 0.285 | 1.16 (0.96–1.41) | 0.144 | 0.250 |
| TNF-beta | 0.93 (0.72–1.20) | 0.570 | 0.644 | 0.98 (0.75–1.28) | 0.871 | 0.871 |

**Statistics:** Data were calculated by Generalized Linear Models (GLM) with a gamma distribution (log-link). Multivariable models were adjusted by age, IL28 genotype, and AST, previously selected by a stepwise method (forward) (see **Results Section**). The q-values represent p-values corrected for multiple testing using the False Discovery Rate (FDR). Significant differences are shown in bold.

**Abbreviations**: AMR, arithmetic mean ratio; aAMR, adjusted AMR; 95%CI, 95% of confidence interval; p, level of significance; q, corrected level of significance; EGF, epidermal growth factor; GRO-alpha/KC, chemokine growth-regulated protein alpha; GM-CSF, granulocyte macrophage colony-stimulating factor; IFN, interferon; IL, interleukin; MCP-1, C-C motif chemokine ligand 2; RANTES, C-C motif chemokine ligand 5; SDF-1alpha, stromal cell-derived factor 1alpha; FGF-2, fibroblast growth factor 2; HGF, hepatocyte growth factor; Beta-NGF, nerve growth factor β; PLGF-1, placental growth factor; SCF, skp, cullin, F-box containing complex; TNF, tumoral necrosis factor.
