# Supplementary material for: Immunological and senescence biomarker profiles in patients after spontaneous clearance of hepatitis C virus: gender implications for long-term health risk
Source: Immun Ageing. 2023 Nov 17;20:62. doi: 10.1186/s12979-023-00387-z (PMC10655350; doi:10.1186/s12979-023-00387-z)
Supplement: Supplementary file 7 — Additional file 7. Comparison of senescence-associated secretory phenotype (SASP) proteins between females who spontaneously cleared HCV (SC group) versus controls (C group). [file 12979_2023_387_MOESM7_ESM.docx]

**Additional File 7.** Comparison of senescence-associated secretory phenotype (SASP) proteins between females who spontaneously cleared HCV (SC group) versus controls (C group).

|  | **Un-adjusted** | | | **Adjusted** | | |
| --- | --- | --- | --- | --- | --- | --- |
| **Marker** | **AMR (95%CI)** | ***p*-value** | ***q*-value** | **aAMR (95%CI)** | ***p*-value** | ***q*-value** |
| EGF | 1.32 (0.97–1.80) | 0.092 | 0.356 | 1.35 (0.98–1.85) | 0.076 | 0.271 |
| Eotaxin | 1.40 (0.88–2.22) | 0.172 | 0.356 | 1.56 (0.96–2.54) | 0.083 | 0.271 |
| Gro-alpha/KC | 1.18 (0.95–1.46) | 0.147 | 0.356 | 1.18 (0.95–1.48) | 0.155 | 0.354 |
| GM-CSF | 1.06 (0.79–1.41) | 0.702 | 0.903 | 1.00 (0.75–1.32) | 0.975 | 0.975 |
| IFN-gamma | 1.02 (0.82–1.26) | 0.878 | 0.913 | 0.97 (0.79–1.19) | 0.784 | 0.886 |
| IL-1beta | 1.03 (0.81–1.32) | 0.790 | 0.903 | 1.05 (0.82–1.34) | 0.710 | 0.879 |
| IL-1alpha | 1.24 (0.96–1.59) | 0.107 | 0.356 | 1.27 (0.99–1.63) | 0.067 | 0.271 |
| IL-1RA | 1.32 (1.03–1.69) | **0.035** | 0.356 | 1.27 (0.99–1.62) | 0.067 | 0.271 |
| IL-2 | 1.01 (0.69–1.47) | 0.957 | 0.957 | 1.04 (0.71–1.52) | 0.860 | 0.894 |
| IL-6 | 0.98 (0.74–1.29) | 0.865 | 0.913 | 1.08 (0.83–1.41) | 0.571 | 0.798 |
| IL-7 | 1.04 (0.79–1.38) | 0.765 | 0.903 | 1.07 (0.82–1.41) | 0.614 | 0.798 |
| IL-8 | 1.19 (0.96–1.49) | 0.130 | 0.356 | 1.18 (0.94–1.49) | 0.163 | 0.354 |
| IL-13 | 1.15 (0.89–1.49) | 0.296 | 0.494 | 1.12 (0.85–1.46) | 0.429 | 0.710 |
| IL-15 | 0.96 (0.73–1.27) | 0.798 | 0.903 | 0.92 (0.71–1.18) | 0.513 | 0.785 |
| IL-18 | 1.25 (0.84–1.86) | 0.288 | 0.494 | 1.23 (0.82–1.87) | 0.329 | 0.611 |
| IP-10 | 1.32 (0.78–2.22) | 0.304 | 0.494 | 1.21 (0.75–1.95) | 0.437 | 0.710 |
| MCP-1 | 1.40 (0.88–2.21) | 0.159 | 0.356 | 1.32 (0.83–2.11) | 0.257 | 0.513 |
| RANTES | 1.07 (0.78–1.45) | 0.691 | 0.903 | 0.97 (0.69–1.35) | 0.841 | 0.894 |
| SDF-1alpha | 1.47 (1.18–1.83) | **0.002** | **0.055** | 1.44 (1.15–1.80) | **0.004** | 0.105 |
| FGF-2 | 1.18 (0.95–1.46) | 0.153 | 0.356 | 1.19 (0.95–1.48) | 0.142 | 0.354 |
| HGF | 1.19 (0.93–1.52) | 0.178 | 0.356 | 1.22 (0.96–1.56) | 0.109 | 0.315 |
| BNGF | 1.18 (0.99–1.39) | 0.072 | 0.356 | 1.20 (1.00–1.42) | **0.048** | 0.271 |
| PIGF-1 | 1.43 (1.01–2.02) | 0.053 | 0.356 | 1.48 (1.04–2.08) | **0.037** | 0.271 |
| SCF | 1.29 (1.00–1.65) | 0.062 | 0.356 | 1.31 (1.02–1.68) | **0.048** | 0.271 |
| TNF-alpha | 1.04 (0.81–1.34) | 0.749 | 0.903 | 1.04 (0.80–1.34) | 0.774 | 0.886 |
| TNF-beta | 1.05 (0.75–1.46) | 0.780 | 0.903 | 1.09 (0.79–1.51) | 0.591 | 0.798 |

**Statistics:** Data were calculated by Generalized Linear Models (GLM) with a gamma distribution (log-link). Multivariable models were adjusted by age, IL28 genotype, and AST, previously selected by a stepwise method (forward) (see **Results Section**). The q-values represent p-values corrected for multiple testing using the False Discovery Rate (FDR). Significant differences are shown in bold.

**Abbreviations**: AMR, arithmetic mean ratio; aAMR, adjusted AMR; 95%CI, 95% of confidence interval; p, level of significance; q, corrected level of significance; EGF, epidermal growth factor; GRO-alpha/KC, chemokine growth-regulated protein alpha; GM-CSF, granulocyte macrophage colony-stimulating factor; IFN, interferon; IL, interleukin; MCP-1, C-C motif chemokine ligand 2; RANTES, C-C motif chemokine ligand 5; SDF-1alpha, stromal cell-derived factor 1alpha; FGF-2, fibroblast growth factor 2; HGF, hepatocyte growth factor; Beta-NGF, nerve growth factor β; PLGF-1, placental growth factor; SCF, skp, cullin, F-box containing complex; TNF, tumoral necrosis factor.
