# Supplementary figures and images for: Immunological and senescence biomarker profiles in patients after spontaneous clearance of hepatitis C virus: gender implications for long-term health risk
Source: Immun Ageing. 2023 Nov 17;20:62. doi: 10.1186/s12979-023-00387-z (PMC10655350; doi:10.1186/s12979-023-00387-z)

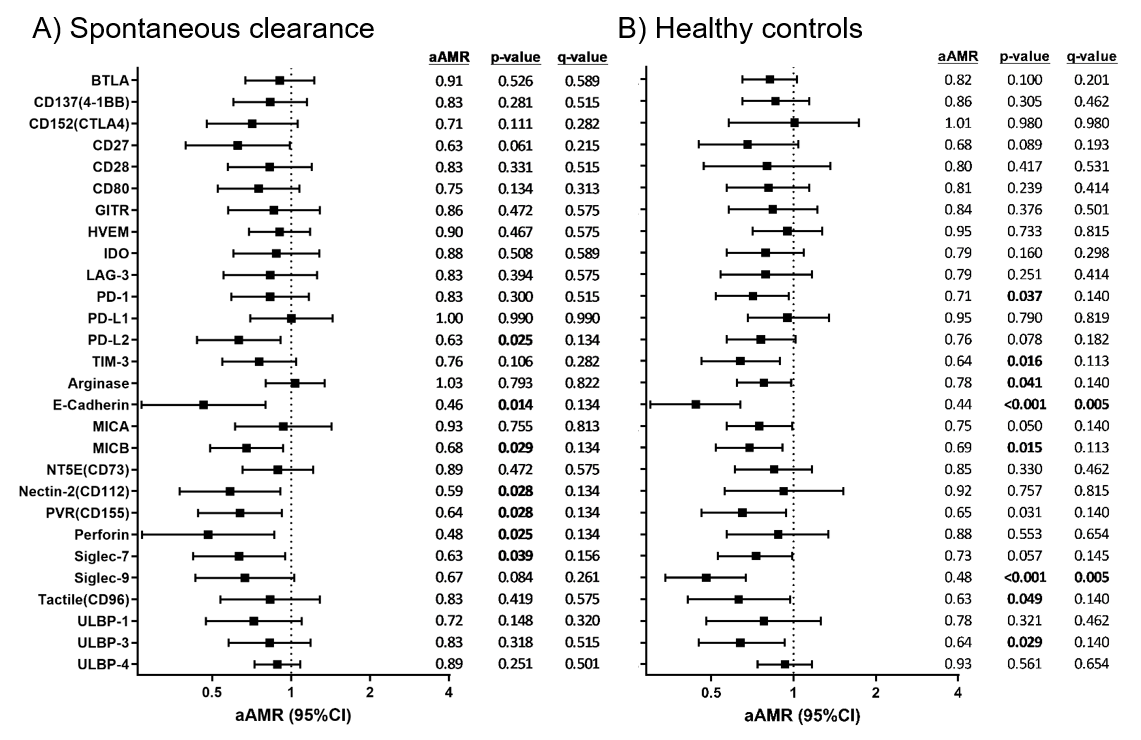

Supplement: Supplementary file 8 — Additional file 8. Comparison of plasma immune checkpoints between sex in both spontaneous clearance (SC) and control (C) groups: A) SC, B) C. [file 12979_2023_387_MOESM8_ESM.tif]

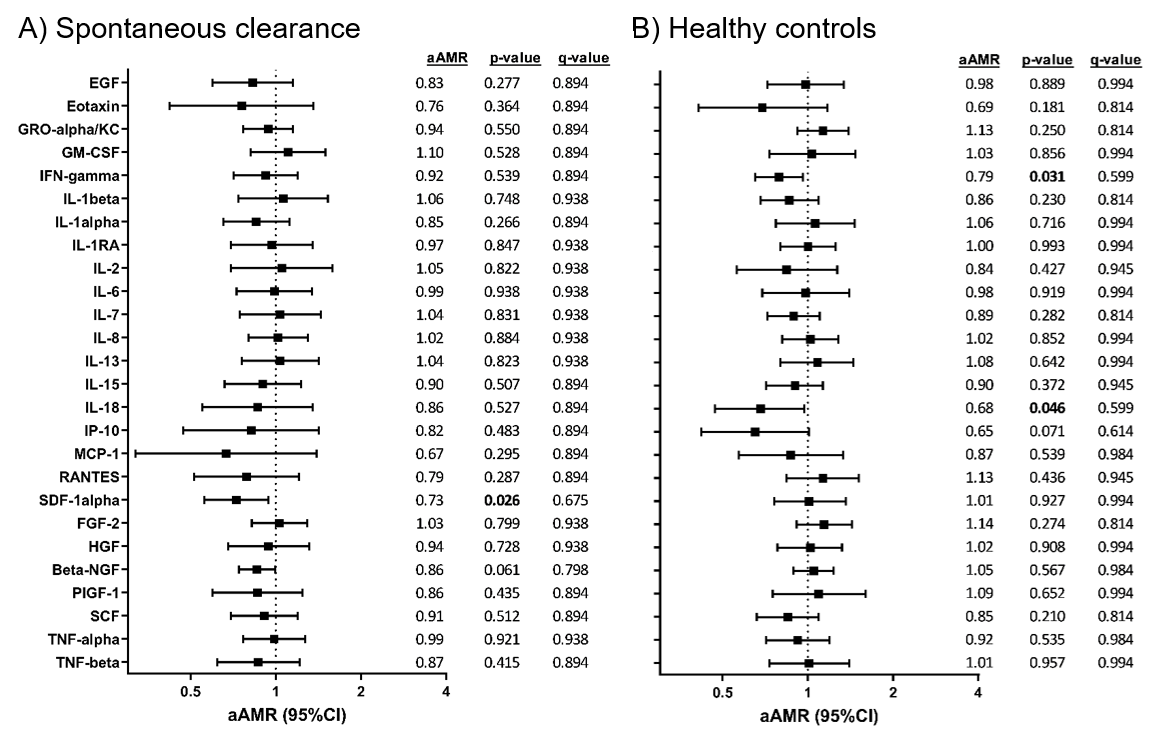

Supplement: Supplementary file 9 — Additional file 9. Comparison of senescence-associated secretory phenotype (SASP) proteins between sex in both spontaneous clearance (SC) and control (C) groups: A) SC, B) C. [file 12979_2023_387_MOESM9_ESM.tif]

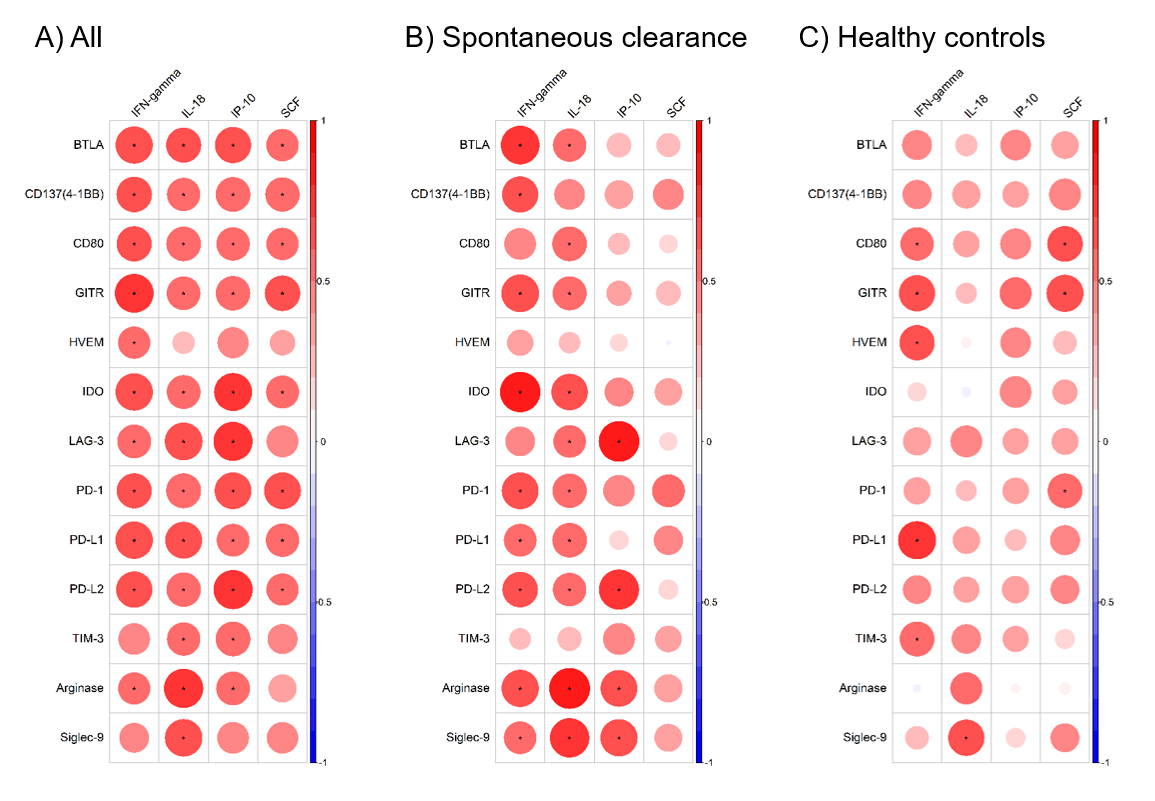

Supplement: Supplementary file 10 — Additional file 10. Spearman correlation plot between significant immune checkpoint and senescence-associated secretory phenotype (SASP) proteins in males: A) ALL males, B) spontaneous clearance (SC), C) control (C) group. [file 12979_2023_387_MOESM10_ESM.tif]
