# Supplementary material for: Immunological and senescence biomarker profiles in patients after spontaneous clearance of hepatitis C virus: gender implications for long-term health risk
Source: Immun Ageing. 2023 Nov 17;20:62. doi: 10.1186/s12979-023-00387-z (PMC10655350; doi:10.1186/s12979-023-00387-z)
Supplement: Supplementary file 11 — Additional file 11. Spearman correlation plot between significant immune checkpoint and senescence-associated secretory phenotype (SASP) proteins in males: A) ALL males, B) spontaneous clearance (SC), C) control (C) group. [file 12979_2023_387_MOESM11_ESM.docx]

**Additional File 11.** Sex determination by PCR.

DNA was extracted with the DNA Purification System Kit (High pure PCR template preparation kit (Roche). The sex chromosome genes *zinc finger protein X-linked* (*ZFX*) and *zinc finger protein Y-linked* (*ZFY*) located on the X and Y chromosomes respectively, were amplified by PCR as previously described by Weiss and Johnston (1999).

Briefly, the PCR mix contain the forward primer ZF_F and two reverse primers specific of the X (ZFX_R) and Y (ZFY_R) chromosomes. Thus, one amplicon of 488 base pairs (bp) (X chromosome specific) is amplified in XX females and two amplicons of 488 and 340 bp (Y chromosome specific) in XY males.

| **Primer** | **Forward sequence (5´-3´)** |
| --- | --- |
| **ZF_F** | ATTGTTCTAAGTCGCCATATTCTCT |
| **ZFX_R** | GAACACACTACTGAGCAAAATGTATA |
| **ZFY_R** | CATCTTTACAAGCTTGTAGACACACT |

The PCR reaction was performed with 2.5µl of 10x Reaction Buffer (Biotools) with a final concentration of 1x, 0.3µM of each of the three primers, 1mM of dNTPs, 2mM of MgCl^2+^, 0.6 U of DNA Polimerase (Biotools) and 50ng of DNA in a final volume of 25ul.

The thermal cycling profile was initiated with 95 ◦C enzyme activation incubation for 3 minutes. Next 35 cycles of 94 ◦C for 15 seconds (s), 60.1 ◦C for 30 s and 72 ◦C for 30 s. After cycling a final elongation was run 7 minutes at 72 ◦C. Sex discrimination was performed by electrophoresis in agarose gels at 2.5%.
